# Robust methods in Mendelian randomization via penalization of heterogeneous causal estimates

**DOI:** 10.1371/journal.pone.0222362

**Published:** 2019-09-23

**Authors:** Jessica M. B. Rees, Angela M. Wood, Frank Dudbridge, Stephen Burgess

**Affiliations:** 1 Cardiovascular Epidemiology Unit, Department of Public Health and Primary Care, University of Cambridge, Cambridge, CB1 8RN, United Kingdom; 2 Edinburgh Clinical Trials Unit, Usher Institute of Population Health Sciences and Informatics, University of Edinburgh, Edinburgh, EH16 4UX, United Kingdom; 3 Department of Health Sciences, University of Leicester, Leicester, LE1 7RH, United Kingdom; 4 MRC Biostatistics Unit, University of Cambridge, Cambridge, CB2 0SR, United Kingdom; Universitair Medisch Centrum Utrecht, NETHERLANDS

## Abstract

Methods have been developed for Mendelian randomization that can obtain consistent causal estimates under weaker assumptions than the standard instrumental variable assumptions. The median-based estimator and MR-Egger are examples of such methods. However, these methods can be sensitive to genetic variants with heterogeneous causal estimates. Such heterogeneity may arise from over-dispersion in the causal estimates, or specific variants with outlying causal estimates. In this paper, we develop three extensions to robust methods for Mendelian randomization with summarized data: 1) robust regression (MM-estimation); 2) penalized weights; and 3) Lasso penalization. Methods using these approaches are considered in two applied examples: one where there is evidence of over-dispersion in the causal estimates (the causal effect of body mass index on schizophrenia risk), and the other containing outliers (the causal effect of low-density lipoprotein cholesterol on Alzheimer’s disease risk). Through an extensive simulation study, we demonstrate that robust regression applied to the inverse-variance weighted method with penalized weights is a worthwhile additional sensitivity analysis for Mendelian randomization to provide robustness to variants with outlying causal estimates. The results from the applied examples and simulation study highlight the importance of using methods that make different assumptions to assess the robustness of findings from Mendelian randomization investigations with multiple genetic variants.

## Introduction

**Fig 1:**
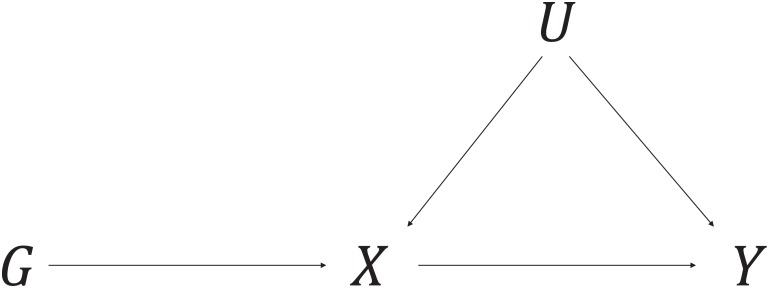
Causal directed acyclic graph illustrating the instrumental variable assumptions for the instrumental variable *G*, exposure *X*, outcome *Y*, and the set of variables (*U*) that confound the association between *X* and *Y*.

Mendelian randomization uses genetic variants as instrumental variables to estimate the causal effect of a risk factor on an outcome using observational data [1, 2]. The genetic variants must satisfy the following criteria (illustrated in Fig 1) to be a valid instrumental variable (IV):

- IV1: the variant is associated with the exposure *X*,
- IV2: the variant is independent of all confounders *U* of the exposure-outcome association, and
- IV3: the variant is independent of the outcome *Y* conditional on the exposure *X* and confounders *U* [3].

A recent development in Mendelian randomization is the availability of summarized data: this consists of the associations (beta-coefficients and standard errors) of genetic variants with the risk factor and with the outcome from regressing each variant in turn [4]. Summarized data can be used to calculate an estimate of the causal effect of the risk factor on the outcome for each genetic variant. The inverse-variance weighted (IVW) method [5] combines these estimates to provide an overall estimate of the causal effect using summarized data from all the genetic variants. If the genetic variants are uncorrelated, the IVW estimate is asymptotically equal to the estimate from the two-stage least squares method commonly used with individual-level data [6].

The inclusion of a variant in a Mendelian randomization analysis that violates either the IV2 or the IV3 assumption may lead to biased causal estimates [7]. Robust methods have therefore been developed to estimate consistent causal effects under weaker assumptions when there are multiple genetic variants. These methods include a median-based method [8] and MR-Egger [9]. Genetic variants that violate the IV assumptions are likely to have heterogeneous causal estimates. We here consider heterogeneity in two settings: firstly, when there is more variance between the variant-specific causal estimates than expected by chance, but the burden of heterogeneity is shared across several genetic variants (over-dispersion); and secondly, when specific variants have outlying causal estimates, and they alone are responsible for driving the observed heterogeneity.

Several robust methods have been proposed that try to identify and remove genetic variants with heterogeneous causal estimates that are suspected to be invalid instruments. These include the MR-PRESSO [10], global and individual tests for direct effects (GLIDE) [11], and generalized summary Mendelian randomization (GSMR) [12] methods. Cochran’s Q-statistic has been used in Mendelian randomization to downweight [8] or exclude genetic variants with heterogeneous causal estimates [13]. The Q-statistic is based on the first order weights of the IVW model which assumes that there is no measurement error (NOME) in the genetic associations with the risk factor [14]. If this assumption is invalid, then the type I error rate of the Q-statistic will be inflated. Bowden *et al*. [14] have accounted for possible violations in the NOME assumption by using adapted second order weights to calculate the Q-statistic.

We here propose three further ways of downweighting or excluding variants with heterogeneous causal estimates that could be considered as part of a sensitivity analysis in a Mendelian randomization study. The first two of these extensions can be used as modifications to either the IVW or the MR-Egger method. These extensions have been influenced by the literature on robust statistics [15], and recent developments in robust methods for Mendelian randomization.

First, we outline the parametric assumptions made throughout the paper and discuss the estimation of the causal effect in a Mendelian randomization study. We then introduce three robust approaches: robust regression (MM-estimation), penalized weights, and Lasso penalization. We apply these approaches to published data on body mass index (BMI) and schizophrenia risk, and on low-density lipoprotein cholesterol (LDL-C) and Alzheimer’s disease (AD) risk. Next, we perform a simulation study under realistic settings to compare bias and coverage properties of the robust methods when some of the genetic variants are invalid IVs. Finally, we discuss the results of the paper and its implications to applied Mendelian randomization research. Software code for implementing all of the methods used in this paper, including extracting the genetic association estimates for the applied examples, is provided in S1 Appendix. The methods (excluding Lasso penalization) can also be applied using the R package *MendelianRandomization* [16].

## Methods

### Parametric assumptions

**Fig 2:**
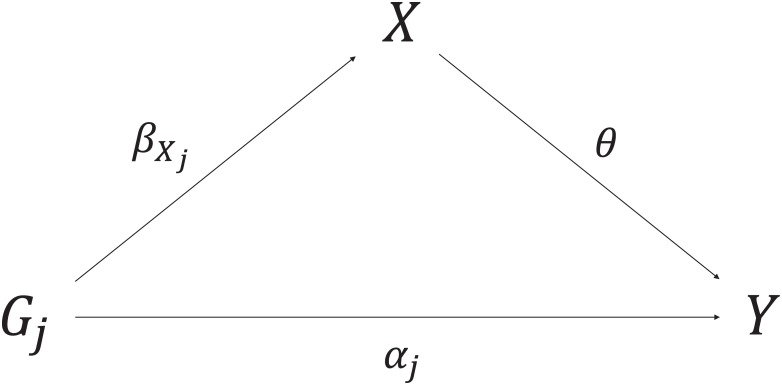
Decomposition of the association between the genetic variant *G*_*j*_ and the outcome *Y* into the indirect effect via the risk factor *X* and direct (pleiotropic) effect *α*_*j*_.

Throughout the paper, we assume linearity and no effect modification of the causal effect *θ* of the risk factor on the outcome, and the associations of the genetic variants *G*_*j*_ (*j* = 1, …, *J*) with the risk factor and with the outcome. These assumptions are not necessary to estimate a causal effect, but they ensure that all valid IVs estimate the same causal parameter. Under these assumptions, the association $\beta_{Y_{j}}$ between the variant *G*_*j*_ and the outcome can be decomposed into an indirect effect via the risk factor and a direct (pleiotropic) effect *α*_*j*_ (illustrated in Fig 2):

$$\begin{array}{c} \beta_{Y_{j}}=\alpha_{j}+\theta \beta_{X_{j}}\;. \end{array}$$ (1)

We also assume that the outcome is a continuous variable. If the outcome is binary, then the methods can be applied to the log odds ratios obtained from logistic regression of each genetic variant on the outcome. The linearity assumption must now hold for the logit-transformed probability of the outcome. Difficulties with interpreting the causal estimate of an odds ratio with a binary outcome and a logistic-linear model have been widely discussed [17], with evidence to suggest that the causal estimates tend to be unbiased under the null [18].

### Estimating the causal effect

The causal effect *θ* can be estimated using the genetic associations with the risk factor (${\hat{\beta }}_{X_{j}}$) and with the outcome (${\hat{\beta }}_{Y_{j}}$). The ratio estimate of the causal effect for variant *j* is given by:

$$\begin{array}{c} {\hat{\theta }}_{j}=\frac{{\hat{\beta }}_{Y_{j}}}{{\hat{\beta }}_{X_{j}}}\;. \end{array}$$ (2)

The *J* ratio estimates can be combined to provide an overall causal estimate by fitting weighted linear regression of the associations of the variants with the outcome on the associations of the variants with the exposure, with the intercept set to zero and $\mathrm{se}{\left({\hat{\beta }}_{Y_{j}}\right)}^{-2}$ as weights:

$$\begin{array}{c} {\hat{\beta }}_{Y_{j}}=\theta \;{\hat{\beta }}_{X_{j}}+\epsilon_{j},\;\epsilon_{j}∼N\left(0,\psi^{2}\mathrm{se}{\left({\hat{\beta }}_{Y_{j}}\right)}^{2}\right)\;. \end{array}$$ (3)

The estimate obtained from Eq (3) is equivalent to the estimate from the IVW method [5]. Under a fixed-effects model, we set the residual standard error (*ψ*) to be equal to one by dividing the standard error of the causal estimate by the estimated residual standard error. To account for heterogeneity (overdispersion) in the causal estimates, the residual standard error can be greater than one under a random-effects model. The causal estimate from the fixed and multiplicative random-effects models will be the same, but the standard error of the causal effect will be larger from the multiplicative random-effects model if there is heterogeneity between the causal estimates.

A genetic variant is pleiotropic if it has a direct effect on the outcome that is not via the risk factor (*α*_*j*_ ≠ 0). The IVW method under a fixed or multiplicative random-effects model will produce a consistent causal estimate when there is no pleiotropy (*α*_*j*_ = 0 for all variants), or when the average pleiotropic effect is zero (referred to as balanced pleiotropy) and the pleiotropic effects are distributed independently of the associations of the genetic variants with the risk factor (known as the InSIDE assumption—Instrument strength independent of the Direct Effect) [9, 19]. If an intercept term in Eq (3) is estimated, then this is the MR-Egger method, and the causal estimate will be consistent in the presence of directional pleiotropy (the average pleiotropic effect differs from zero) if the InSIDE assumption is satisfied [9]:

$$\begin{array}{c} {\hat{\beta }}_{Y_{j}}=\theta_{0}+\theta_{1}\;{\hat{\beta }}_{X_{j}}+\epsilon_{j},\;\epsilon_{j}∼N\left(0,\psi_{E}^{2}\mathrm{se}{\left({\hat{\beta }}_{Y_{j}}\right)}^{2}\right)\;. \end{array}$$ (4)

If the genetic variants are all valid IVs, then the ratio estimates for each variant should be similar. If the ${\hat{\beta }}_{Y_{j}}$ estimates were plotted against the ${\hat{\beta }}_{X_{j}}$ estimates, a pleiotropic variant may appear as an outlier relative to the valid IVs as the direct effect of the pleiotropic variant will result in the vertical displacement of ${\hat{\beta }}_{Y_{j}}$ from the causal effect (Eq (1)). Robust methods that downweight the contribution of variants with heterogeneous ratio estimates should reduce the impact that variants with outlying or over-dispersed estimates have on the causal estimate. For example, the simple median estimator is the median of the *J* ratio estimates *θ*_*j*_ (*j* = 1, …, *J*), and will produce consistent causal estimates if at least 50% of the genetic variants are valid IVs [8].

Typically, applied Mendelian randomization analyses will use one variant from each gene region. Under Mendel’s second law, these variants should be independently distributed due to their physical separation. The methods discussed in this paper will therefore assume that the variants are uncorrelated.

#### Robust regression (MM–estimation)

The breakdown point is a measure of the robustness of an estimator to contaminations (such as outliers) in the dataset [15]. Ordinary least squares (OLS) has a breakdown point of 0% as all of the observations have equal weight and just one outlying observation can heavily influence the estimator, resulting in an arbitrarily large or small estimate. Robust regression methods, such as MM-estimation, have been proposed where the breakdown point is greater than 0% [15].

In this paper, we use an MM-estimation approach proposed by Koller and Stahel [20] as it retains the high asymptotic efficiency of the M-estimator (‘maximum likelihood type’), whilst utilising the S-estimator (‘scale-type estimate’) to provide robustness against outliers and leverage points. Under this method, a S-estimate is fitted to minimize the M-estimate of scale, which has the desired high breakdown point but may lack efficiency. The estimates for the scale and regression parameters obtained in this stage are then used to fit an M-estimator with high efficiency, where the scale estimate is held constant to retain the high-breakdown point [20].

Additional robustness in MM-estimation may be achieved by using Tukey’s bisquare objective function in the estimation procedure with its weighting function:

$$\begin{array}{c} w\left(r_{j}\right)=\{\begin{array}{cc} {\left[1-{\left(\frac{r_{j}}{c}\right)}^{2}\right]}^{2}\; & \text{if}\;|r_{j}|<c\\ 0\; & \text{if}\;|r_{j}|\geq c \end{array}\;, \end{array}$$

where *r*_*j*_ are the standardized residuals, and *w*(*r*_*j*_) are used in the objective function of the iteratively reweighted least squares algorithm to obtain the MM-estimates. The recommended values for the tuning parameter *c* maintain a high breakdown point in the S-estimation step (*c* = 1.548) and provide efficiency in the M-estimation step (*c* = 4.685). In MM-estimation with Tukey’s bisquare objective function, the weight of an observation decreases as *r*_*j*_ tends away from zero, and when |*r*_*j*_| ≥ *c* the observation will have zero weight.

Throughout the paper, we will refer to this approach as robust regression. It is the default implementation of robust regression for the lmrob command in the R package *robustbase* [21]. Since the lmrob command allows the user to specify a vector of weights to be used in conjunction with Tukey’s weighting function, robust regression can be used instead of ‘ordinary regression’ (weighted least squares) for the IVW and MR-Egger methods.

#### Penalized weights

We assume that the NOME assumption is satisfied, and propose an approach for downweighting genetic variants with heterogeneous ratio estimates in the IVW model using Cochran’s Q statistic:

$$\begin{array}{c} Q=\sum_{j}Q_{j}=\sum_{j}\mathrm{se}{\left({\hat{\beta }}_{Y_{j}}\right)}^{-2}{\left({\hat{\beta }}_{Y_{j}}-\hat{\theta }{\hat{\beta }}_{X_{j}}\right)}^{2}\;, \end{array}$$ (5)

which has an approximate $\chi_{J-1}^{2}$ distribution under the null hypothesis that all *J* genetic variants satisfy the IV assumptions, with the *J* components *Q*_*j*_ (*j* = 1, …, *J*) having approximate $\chi_{1}^{2}$ distributions [13]. Since penalized weights would normally be considered when pleiotropy is suspected, the simple (unweighted) median estimate is used for the value of $\hat{\theta }$ in Eq (5) rather than the IVW estimate.

To ensure that the weights ($\mathrm{se}{\left({\hat{\beta }}_{Y_{j}}\right)}^{-2}$) for the majority of the variants remain the same, we use a penalization for the IVW method based on the one-sided upper tail probability (denoted *q*_*j*_) of *Q*_*j*_ on a $\chi_{1}^{2}$ distribution by multiplying the weights by min(1, 100*q*_*j*_). A similar downweighting factor, min(1, 20*q*_*j*_), was used for the penalized–median estimator in the paper by Bowden *et al*. [8]. Initially we used min(1, 20*q*_*j*_) but found that too many variants were being penalized, resulting in over-precise estimates that had poor coverage of the true causal effect. By multiplying the weights by min(1, 100*q*_*j*_), the outlying variants should be severely penalized, without downweighting too many genetic variants that are valid IVs.

For the MR-Egger method, we consider the modified Q’ statistic [22]:

$$\begin{array}{c} Q^{\prime }=\sum_{j}Q_{j}^{\prime }=\sum_{j}\mathrm{se}{\left({\hat{\beta }}_{Y_{j}}\right)}^{-2}{\left({\hat{\beta }}_{Y_{j}}-{\hat{\theta }}_{0}-{\hat{\theta }}_{1}{\hat{\beta }}_{X_{j}}\right)}^{2}\;, \end{array}$$ (6)

where ${\hat{\theta }}_{0}$ and ${\hat{\theta }}_{1}$ are taken from the MR-Egger model. If the MR-Egger model is correct, the Q’ statistic in Eq (6) should follow an approximate $\chi_{J-2}^{2}$ distribution [23]. The penalized weights described in this Section can also be applied to robust regression for the IVW and MR-Egger methods, subsequently referred to as the robust and penalized approach (or robust regression with penalized weights).

#### Lasso penalization

The application of Lasso regression in IV analyses has already been considered in the literature [24–26]. The penalty term in Lasso regression shrinks the regression coefficients towards zero, and forces some coefficients to be zero [27]. The sparsity property (shrinking some coefficients to zero) of Lasso regression has been used to identify and remove invalid IVs. The IV methods that use Lasso regression have only been considered with respect to individual level data.

We take the ‘post-lasso’ method proposed by Windmeijer *et al*. [25] for individual level data and adapt this method to be used with summary level data. First, we consider the objective function for the MR-Egger model that is minimized when fitting the regression model of Eq (4):

$$\begin{array}{c} \sum_{j}\mathrm{se}{\left({\hat{\beta }}_{Y_{j}}\right)}^{-2}{\left({\hat{\beta }}_{Y_{j}}-\theta_{0}-\theta_{1}{\hat{\beta }}_{X_{j}}\right)}^{2}\;. \end{array}$$

To better model the pleiotropic effects *α*_*j*_ in Eq (1), we propose replacing *θ*_0_ with a separate intercept coefficient for each genetic variant $\theta_{0_{j}}$, and adding a Lasso-penalty term for the $\theta_{0_{j}}$ parameters:

$$\begin{array}{c} \sum_{j}\mathrm{se}{\left({\hat{\beta }}_{Y_{j}}\right)}^{-2}{\left({\hat{\beta }}_{Y_{j}}-\theta_{0_{j}}-\theta_{1}{\hat{\beta }}_{X_{j}}\right)}^{2}+\lambda \sum_{j}\left|\theta_{0_{j}}\right|\;. \end{array}$$ (7)

If $\theta_{0_{j}}$ shrinks to zero in Eq (7), the genetic variant is treated as a valid IV. We take the genetic variants with a zero intercept term $\theta_{0_{j}}$, and perform the IVW method using these variants only to estimate the causal effect *θ*. The degree of shrinkage in Eq (7) is determined by the value of the tuning parameter λ. If λ = ∞, then all of the genetic variants are assumed to be valid instruments as $\theta_{0_{j}}$ is forced to be zero for all *J* variants, and the IVW method is performed using the full set of genetic variants. If λ = 0, then all of the variants can be pleiotropic, and the parameters in Eq (7) are not identified.

To determine the value of λ, two rules were considered: 1) a heterogeneity stopping rule; and 2) a cross-validation rule. The heterogeneity stopping rule is influenced by the method used by Windmeijer *et al*. [25] and Cochran’s Q statistic. For the heterogeneity stopping rule, we fit the Lasso penalization model (Eq (7)) over a range of values for λ, starting with a value close to zero, and then increasing λ in small increments. We stop at λ = λ_*n*_ when the residual standard error from the IVW model, based on the variants determined to be valid from λ = λ_*n*+1_, is greater than 1, and the increase in the residual standard error from λ_*n*_ to λ_*n*+1_ is greater than $\chi_{1}^{2}\left(0.95\right)/J_{inc}$, where $\chi_{1}^{2}\left(0.95\right)$ is the upper 95th percentile of a chi-squared distribution on 1 degree of freedom, and *J*_*inc*_ is the number of genetic variants included in the IVW model when λ = λ_*n*+1_.

As an alternative to the heterogeneity stopping rule, we use the optL1 command in the R package *penalized* [28]. optL1 compares the predictive ability of the Lasso regression model for different values of λ through leave-one-out cross-validation. The optimal value of λ is then determined by maximizing the cross-validated likelihood function.

### Summary

In this Section, we have introduced three robust approaches that can be used in a Mendelian randomization study as part of the sensitivity analysis. The approaches use summary level data that either downweight or remove genetic variants that have heterogeneous causal ratio estimates. In the next Section, we apply these approaches to published summary data to investigate the causal effect of body mass index on schizophrenia risk, and the causal effect of low-density lipoprotein cholesterol on Alzheimer’s disease risk.

## Applied examples

To illustrate the performance of the proposed extensions, we considered two applied examples: one where there was evidence of over-dispersion in the ratio estimates (the causal effect of BMI on schizophrenia risk); and another that contained outliers (the causal effect of LDL-C on AD risk). Using summary data (beta–coefficients and standard errors) from PhenoScanner [29], we considered the IVW method with: 1) the full set of genetic variants; 2) robust regression; 3) penalized weights; and 4) robust regression and penalized weights. Lasso penalization with the heterogeneity stopping and cross-validation rules, the simple median, the weighted median, and the MR-Egger methods were also considered. Under the heterogeneity stopping rule, the Lasso penalization model was applied to λ = 0.1, 0.2, …, 4.9, 5.0, 5.2, 5.4, …, 9.8, 10.0. Multiplicative random-effects models were used in all analyses.

### Causal effect of body mass index on schizophrenia risk

Although individuals with schizophrenia tend to be overweight [30], it is generally believed that this is due to the effect of anti-psychotic medication on body composition (reverse causation) rather than any causal effect of BMI on schizophrenia risk [31]. For this Mendelian randomization analysis, we used the 97 genetic variants reported by the Genetic Investigation of Anthropometric Traits (GIANT) consortium that were associated with BMI in 339,224 European-descent individuals at a genome-wide level of significance (p-value < 5 × 10^−8^) [32]. Variants were clumped at a correlation threshold of *r*^2^ > 0.1, and all 97 variants are separated by at least 500 kilobases. The genetic associations with schizophrenia were obtained from the Psychiatric Genomics Consortium (PGC) based on 35,476 cases and 46,839 controls mostly of European descent [33]. The summarized data used in this paper were recently applied in a Mendelian randomization study investigating the causal effect of BMI on psychiatric disorders, including schizophrenia risk [34].

### Causal effect of low-density lipoprotein cholesterol on Alzheimer’s disease risk

Epidemiological studies have provided evidence of an association between LDL-C and increased risk of AD [35, 36]. However, there is also evidence to suggest that patients with AD have altered lipid metabolism (reverse causation) [37]. In this Mendelian randomization analysis, we used the 75 genetic variants previously demonstrated to be associated with LDL-C at a genome-wide level of significance by the Global Lipids Genetics Consortium (GLGC) [38]. The point estimates for the genetic associations with LDL-C were taken from the linear regression in up to 188,578 participants from GLGC [39]. The majority of variants are separated by at least 1 megabase. A second variant from a gene region was only selected if it was independently associated with LDL-C and in low linkage disequilibrium with the lead variant (*r*^2^ < 0.05). A recent Mendelian randomization study used summarized data from GLGC to investigate the causal association between low LDL-C levels and AD risk using data on 380 variants. Our analysis is based on a smaller set of genetic variants compared to Benn *et al*. [40] as we excluded variants that were associated with LDL-C and high-density lipoprotein and/or triglycerides. The genetic associations with AD were obtained from the International Genomics of Alzheimer’s Project (IGAP) based on 17,008 cases and 37,154 controls of European-descent [41].

### Results

**Fig 3:**
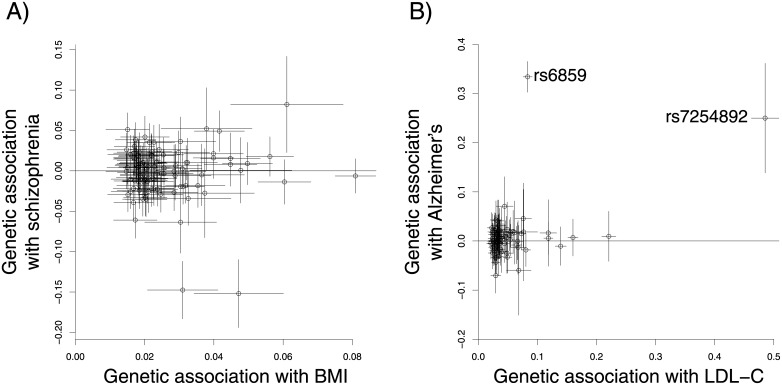
Graph A) displays the estimated genetic associations and 95% confidence intervals with body mass index (BMI, standard deviation units) and with schizophrenia (log odds ratios) for 97 genetic variants. Graph B) displays the estimated genetic associations and 95% confidence intervals with low-density lipoprotein cholesterol (LDL-C, standard deviation units) and with Alzheimer’s disease (log odds ratios) for 75 genetic variants: the two outlying variants are labelled with their rsID codes.

The estimated genetic associations with 95% confidence intervals for the two examples are displayed in Fig 3. The plots demonstrate the overdispersion in the ratio estimates for BMI and schizophrenia; and two outliers in the LDL-C and AD example. The outlying variants (rs6859 and rs7254892) for LDL-C and AD are located near to the *APOE* locus and are associated with AD risk with odds ratios of 1.40 (95% CI: 1.35, 1.44) and 1.28 (95% CI: 1.15, 1.44) respectively [41]. Studentized residuals from the IVW analysis for these variants are 16.5 and −0.95 (all other variants had absolute Studentized residual less than 2), and Cook’s distances are 2.51 and 0.11 respectively (all other Cook’s distances were less than 0.06).

Estimates and 95% confidence intervals from the Mendelian randomization analyses are provided in Table 1. All of the estimates for BMI and schizophrenia suggest a null causal effect (as also observed in the Mendelian randomization study by Hartwig *et al*. [34]), although there is wide variation in the standard errors. The use of penalized weights and robust regression in the IVW method improved the precision of the estimates. There was little difference in the point estimates or standard errors obtained from the IVW method with penalized weights, and from the IVW method with robust regression and penalized weights. With exception of the IVW and MR-Egger methods, the median estimates were the least precise.

**Table 1:** Estimates (standard errors) and 95% confidence intervals of the causal effect of body mass index on schizophrenia risk (log odds ratio for schizophrenia per 1 standard deviation increase in body mass index) and low-density lipoprotein cholesterol on Alzheimer’s disease risk (log odds ratio for Alzheimer’s per 1 standard deviation increase in low-density lipoprotein cholesterol) from the IVW method with: 1) the full set of genetic variants (IVW); 2) robust regression; 3) penalized weights; and 4) robust regression and penalized weights. Results from Lasso penalization with the heterogeneity stopping rule and cross-validation, simple median, weighted median and MR-Egger methods are also presented.

	Estimate (SE)	95% CI
**Applied example 1: Causal effect of BMI on schizophrenia risk**		
IVW	-0.031 (0.100)	-0.227, 0.165
Robust regression	-0.024 (0.079)	-0.180, 0.132
Penalized weights	-0.056 (0.065)	-0.184, 0.073
Robust regression with penalized weights	-0.052 (0.066)	-0.182, 0.078
Lasso penalization		
Heterogeneity stopping rule	-0.022 (0.055)	-0.131, 0.086
Cross validation	-0.036 (0.087)	-0.207, 0.136
Median		
Simple	-0.073 (0.083)	-0.237, 0.090
Weighted	-0.075 (0.090)	-0.252, 0.102
MR-Egger	0.336 (0.241)	-0.136, 0.808
**Applied example 2: Causal effect of LDL-C on AD risk**		
IVW	0.239 (0.102)	0.039, 0.439
Robust regression	0.048 (0.038)	-0.027, 0.123
Penalized weights	0.040 (0.042)	-0.043, 0.123
Robust regression with penalized weights	0.046 (0.032)	-0.016, 0.108
Lasso penalization		
Heterogeneity stopping rule	0.032 (0.044)	-0.054, 0.118
Cross validation	0.088 (0.045)	0.000, 0.175
Median		
Simple	0.108 (0.071)	-0.031, 0.247
Weighted	0.046 (0.061)	-0.073, 0.165
MR-Egger	0.391 (0.168)	0.061, 0.722

Abbreviations: SE, standard error; CI, confidence interval; BMI, body mass index; IVW, inverse-variance weighted; LDL-C, low-density lipoprotein cholesterol; AD, Alzheimer’s disease.

**Fig 4:**
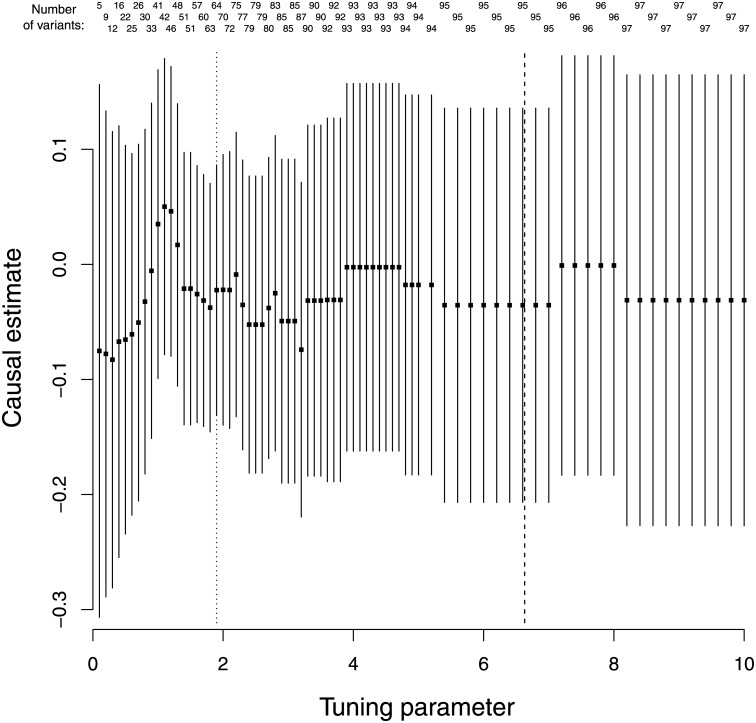
Log odds ratio and 95% confidence intervals for schizophrenia per 1 standard deviation increase in body mass index for different values of the tuning parameter (λ = 0.1, 0.2, …, 4.9, 5.0, 5.2, 5.4, …, 9.8, 10.0) included in the Lasso regression model. The number of genetic variants included in the IVW models are also displayed. The dotted line at λ = 1.9 is the value of the tuning parameter chosen by the heterogeneity stopping rule. The dashed line at λ = 6.63 is the value chosen by cross-validation.

The Lasso penalization estimates are displayed in Fig 4 where the causal estimates are relatively similar across the different values of the tuning parameter. The value of the tuning parameter λ was 1.9 under the heterogeneity stopping rule, with 64 genetic variants included in the IVW method. The cross-validation method returned a much larger value of λ = 6.63, with 95 of the 97 variants included in the IVW method.

The estimates from the IVW and MR-Egger methods suggested a positive causal effect of LDL-C on AD risk. This effect was attenuated to the null for the other robust methods. Compared to the robust methods that reported a null causal effect of LDL-C on AD risk, the simple and weighted median estimates had larger standard errors. The estimates from the IVW and MR-Egger methods from Benn *et al*. [40] indicated that lower LDL-C levels may be beneficial in reducing AD risk, whereas their estimate from the weighted median method suggested a null effect. Since the genetic variants in the *APOE* gene region tend to be highly pleiotropic [10], it is likely that the positive effects obtained from the IVW models in our analysis and in the paper by Benn *et al*. [40] are driven by these pleiotropic variants, rather than there being a true causal effect of LDL-C on AD risk.

**Fig 5:**
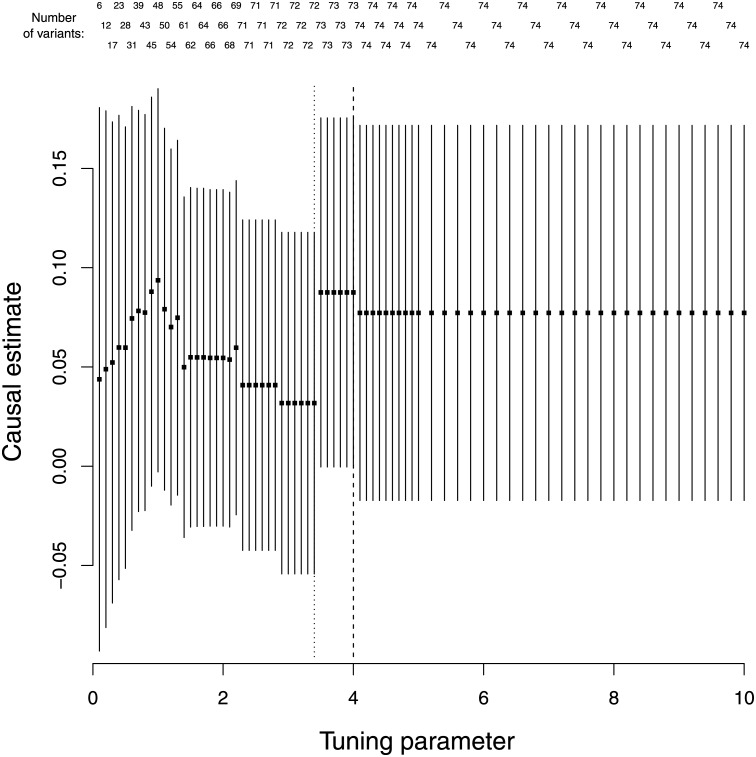
Log odds ratio and 95% confidence intervals for Alzheimer’s per 1 standard deviation increase in low-density lipoprotein cholesterol for different values of the tuning parameter (λ = 0.1, 0.2, …, 4.9, 5.0, 5.2, 5.4, …, 9.8, 10.0) included in the Lasso regression model. The number of genetic variants included in the IVW models are also displayed. The dotted line at λ = 3.4 is the value of the tuning parameter chosen by the heterogeneity stopping rule. The dashed line at λ = 4.00 is the value chosen by cross-validation.

The λ values for the heterogeneity stopping rule (λ = 3.4 based on 72 genetic variants) and cross-validation (λ = 4.00 based on 73 genetic variants) for Lasso penalization were similar (Fig 5). However, the estimate based on 72 genetic variants was much closer to the null, demonstrating the sensitivity of the IVW method to a single variant. None of the estimates in Fig 5 include information on the rs6859 variant, and this outlying variant was only included in the IVW model for Lasso penalization when λ = 19.8, whereas the other outlying variant (rs57254892) was included when λ = 3.5.

The consistency of the results from the robust methods for the BMI and schizophrenia example strengthened the evidence from the primary IVW analysis, providing similar point estimates but with narrower confidence intervals. The LDL-C and AD example highlighted the possibility that only using the IVW method may provide conclusions that are not representative of the majority of the data. Whilst in practice the outlying rs6859 variant could have been identified and removed from the dataset prior to the analysis, the robust approaches identified this outlying variant in an automated manner.

## Simulation study

### Approaches applied to the simulated data

We applied the approaches introduced in this paper to simulated datasets, including the IVW method with: 1) all the *J* genetic variants (standard IVW method); 2) robust regression; 3) penalized weights; and 4) robust regression and penalized weights. The Lasso penalization method with the heterogeneity stopping rule was also considered. The bias and coverage properties of the estimates from these robust methods were compared to those from the simple (unweighted) median, weighted median, and MR-Egger methods. Standard errors for the simple and weighted median estimates were obtained through bootstrapping [8]. Robust regression, penalized weights, and robust regression and penalized weights were also applied to the MR-Egger model. The Lasso penalization method was applied to λ = 0.1, 0.2, …, 4.9, 5.0, 5.2, 5.4, …, 9.8, 10.0 under the heterogeneity stopping rule.

To allow for direct comparisons with the MR-Egger method, and to assess the performance of the methods when the IV assumptions were violated, the simulations followed a similar structure to the simulation study performed in the paper by Bowden *et al*. [8]. The data generating model used in the simulation study is outlined below.

### Data generating model

**Fig 6:**
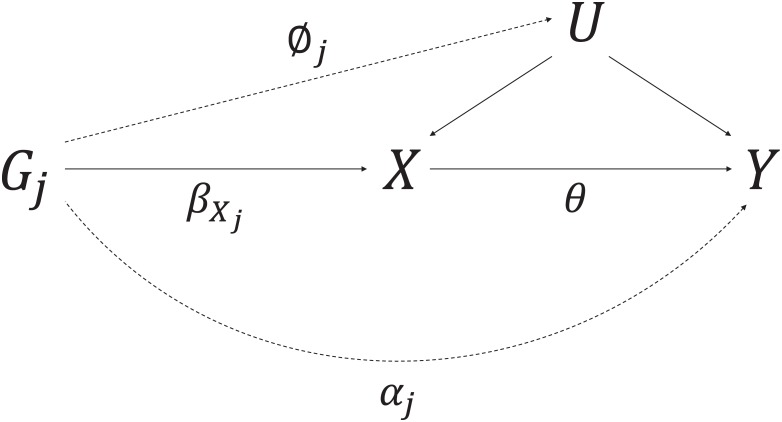
Causal directed acyclic graph used in the data generating model for the simulation study. *U* represents the set of variables that confound the association between the risk factor *X* and outcome *Y*. The genetic effect of *G*_*j*_ on *X* is $\beta_{X_{j}}$, the direct (pleiotropic) effect of *G*_*j*_ on *Y* is *α*_*j*_, the effect of *G*_*j*_ on *U* is *ϕ*_*j*_, and the causal effect of *X* on *Y* is *θ*.

The simulation study generated data in accordance to Fig 6 for participants indexed by *i* = 1, …, *N*, and genetic variants indexed with *j* = 1, …, *J*:

$$\begin{array}{cc} U_{i} & =\sum_{j=1}^{J}\phi_{j}G_{ij}+\epsilon_{Ui}\;,\\ X_{i} & =\sum_{j=1}^{J}\beta_{X_{j}}G_{ij}+U_{i}+\epsilon_{Xi}\;,\\ Y_{i} & =\sum_{j=1}^{J}\alpha_{j}G_{ij}+\theta X_{i}+U_{i}+\epsilon_{Yi}\;,\\ G_{ij} & ∼\text{Binomial}(2,0.3)\;\text{independently}\;\text{for}\;\text{all}\;j=1,\ldots ,J\;,\\ \epsilon_{Ui},\epsilon_{Xi},\epsilon_{Yi} & ∼N(0,1)\;\text{independently}\;, \end{array}$$

where *α*_*j*_ represents the direct effect of the genetic variant *G*_*j*_ on the outcome, *ϕ*_*j*_ represents the effect of the genetic variant on the confounder *U* of the risk factor *X* and outcome *Y* association, $\beta_{X_{j}}$ represents the genetic effect of *G*_*j*_ on *X*, and *θ* is the causal effect of *X* on *Y*. The error terms *ϵ*_*Ui*_, *ϵ*_*Xi*_, and *ϵ*_*Yi*_ were drawn independently from standard normal distributions.

The performance of the robust methods was investigated under a two-sample Mendelian randomization setting with *N* = 10, 000 individuals and *J* = 15 genetic variants. Data were generated for 2*N* participants, and the associations of the variants with the risk factor were estimated in the first *N* participants, and associations with the outcome in the second *N* participants. Only the summary level data (beta-coefficients and standard errors) were used in the analyses. A one-sample setting was also considered where an additional *N* participants were simulated and all of the genetic associations were estimated from the same *N* participants.

If a genetic variant is associated with a confounder of the risk factor–outcome association, then this will affect the variant’s association with both the risk factor and the outcome, leading to the violation of the InSIDE assumption. Using this observation, data were simulated to consider the following four scenarios:

- Scenario 1—No pleiotropy, InSIDE automatically satisfied: *α*_*j*_ and *ϕ*_*j*_ were set to zero for all *j*.
- Scenario 2—Balanced pleiotropy, InSIDE satisfied: *α*_*j*_ ∼ *U*[0.05, 0.15] for invalid variants, with each *α*_*j*_ having a 0.5 probability of being multiplied by -1. *ϕ*_*j*_ was set to zero for all *j*.
- Scenario 3—Directional pleiotropy, InSIDE satisfied: *α*_*j*_ ∼ *U*[0.05, 0.15] for invalid variants, and *ϕ*_*j*_ was set to zero for all *j*.
- Scenario 4—Directional pleiotropy, InSIDE violated: *ϕ*_*j*_ ∼ *U*[0.05, 0.10] for invalid variants, and *α*_*j*_ was set to zero for all *j*.

The genetic variants *G*_*j*_ were coded to correspond to a single nucleotide polymorphism with minor allele frequency 0.3. If a genetic variant was a valid IV then *α*_*j*_ and *ϕ*_*j*_ were set to zero in all four scenarios. In Scenarios 2 to 4, the number of invalid IVs was set to 1, 3 and 6. The causal effect of the risk factor on the outcome was either *θ* = 0 (null causal effect) or *θ* = 0.3 (positive causal effect). The effects of the genetic variants on the risk factor ($\beta_{X_{j}}$) were drawn from a uniform distribution between 0.06 and 0.13. 10 000 simulated datasets were generated for each combination of parameters (24 different combinations in total).

### Results

The mean proportion of variance in the risk factor explained by the genetic variants (R^2^ statistic), mean F statistic, and mean *I*^2^ statistic are contained in Table A in the S2 Appendix for scenarios 1-4 for the null and positive causal effects by the number of invalid instruments. The mean R^2^ values were greater than 3% for all of the scenarios, and the minimum mean F-statistic was 20.8. The *I*^2^ statistic ranged from 39.1% to 80.9%. Since violations in the no measurement error (NOME) assumption of the genetic associations with the risk factor can lead to attenuation towards the null for the MR-Egger estimates, and this attenuation is approximately equal to the *I*^2^ statistic, we expected the MR-Egger estimates for the positive causal effect to be severely attenuated towards the null [42].

The number of robust regression models that did not report a standard error (maximum of 2.6% across all of the scenarios considered) are given in Table B in the S2 Appendix. Apart from the calculation of the mean standard error, the robust regression models that did not report a standard error were included in the results, and the power calculations treated the standard error as infinite.

When all of the genetic variants were valid IVs (Table 2), all of the methods produced unbiased estimates of the null causal effect and the Type I error rates were close to the nominal level of 5%. Apart from the simple median method, there was attenuation towards the null with a positive causal effect for all methods, and as expected, this was particularly evident for the MR-Egger method (also observed for Scenarios 2 and 3). Violation of the NOME assumption can lead to inflation of the intercept term in the MR-Egger method [42], and this was true for the simulation study where the power to detect the intercept term for Scenarios 1 and 2 was greater than 5% (Table C in the S2 Appendix). Only 7.5% of the MR-Egger models detected a positive causal effect, and apart from the median estimators, all of the robust methods had approximately 95% power to detect the positive causal effect.

**Table 2:** Mean estimate (mean standard error), standard deviation, coverage of the 95% confidence interval (%), and power at the 5% significance level (%) of the estimates from the IVW model with: 1) the *J* genetic variants (IVW); 2) robust regression; 3) penalized weights; and 4) robust regression and penalized weights for Scenario 1 with a null (*θ* = 0) or positive (*θ* = 0.3) causal effect. Results from Lasso penalization with the heterogeneity stopping rule, simple (unweighted) median, weighted median and MR-Egger methods are also provided.

	Null causal effect (*θ* = 0)				Positive causal effect (*θ* = 0.3)			
	Estimate (SE)	SD	Cov.	Pow.	Estimate (SE)	SD	Cov.	Pow.
**Scenario 1**. No pleiotropy, InSIDE automatically satisfied								
IVW	-0.001 (0.061)	0.058	95.7	4.3	0.287 (0.073)	0.069	95.5	98.2
Robust regression	-0.001 (0.066)	0.060	95.1	4.9	0.287 (0.079)	0.072	94.7	94.8
Penalized weights	-0.001 (0.060)	0.059	95.0	5.0	0.289 (0.072)	0.071	94.7	98.2
Robust regression with penalized weights	-0.001 (0.064)	0.061	94.5	5.5	0.288 (0.077)	0.073	94.1	95.7
Lasso penalization	-0.001 (0.060)	0.059	94.8	5.2	0.287 (0.072)	0.071	94.6	98.0
Median								
Simple	-0.002 (0.086)	0.074	97.9	2.1	0.301 (0.105)	0.090	98.0	86.9
Weighted	-0.002 (0.080)	0.071	97.4	2.6	0.277 (0.097)	0.085	96.7	85.7
MR-Egger	-0.001 (0.219)	0.207	96.1	3.9	0.143 (0.261)	0.251	91.0	7.5

Abbreviations: SE, standard error; SD, standard deviation; Cov., coverage; Pow., power; InSIDE, instrument strength independent of direct effect; IVW, inverse-variance weighted.

Although the mean estimates in Scenario 2 (Tables 3 and 4) were similar to those in Scenario 1, there were clear differences in the precision of the estimates for the null and positive causal effects, with most of the methods reporting larger mean standard errors under Scenario 2. The mean standard error increased as the number of invalid instruments increased for all methods. The IVW model with penalized weights had the most precise estimates, but suffered from inflated Type I error rates and poor coverage. The simple and weighted median estimators performed just as well, if not better, than the other robust methods for Scenario 2.

**Table 3:** Mean estimate (mean standard error), standard deviation, coverage of the 95% confidence interval (%), and power at the 5% significance level (%) of the estimates from the IVW model with: 1) the *J* genetic variants (IVW); 2) robust regression; 3) penalized weights; and 4) robust regression and penalized weights (R and P) for Scenarios 2-4 with a null causal effect (*θ* = 0) by the number of invalid IVs. Results from Lasso penalization with the heterogeneity stopping rule, simple median, weighted median and MR-Egger methods are also provided.

	1 invalid IV				3 invalid IVs				6 invalid IVs			
	Est. (SE)	SD	Cov.	Pow.	Est. (SE)	SD	Cov.	Pow.	Est. (SE)	SD	Cov.	Pow.
**Scenario 2**. Balanced pleiotropy, InSIDE satisfied												
IVW	-0.002 (0.089)	0.092	94.7	5.3	0.000 (0.133)	0.136	93.4	6.6	0.000 (0.180)	0.183	93.0	7.0
Robust	-0.002 (0.069)	0.065	94.3	5.7	0.000 (0.096)	0.087	94.5	5.5	0.001 (0.196)	0.173	94.3	5.6
Penalized	-0.002 (0.062)	0.064	94.2	5.8	0.000 (0.066)	0.077	91.1	8.9	0.001 (0.075)	0.116	81.5	18.5
R and P	-0.002 (0.071)	0.065	94.6	5.4	0.001 (0.094)	0.078	94.7	5.2	0.001 (0.160)	0.119	91.5	7.3
Lasso	-0.002 (0.063)	0.065	94.4	5.6	0.000 (0.071)	0.080	91.7	8.3	0.001 (0.088)	0.129	84.5	15.5
Median												
Simple	-0.002 (0.090)	0.080	97.4	2.6	0.001 (0.097)	0.094	96.5	3.5	0.002 (0.115)	0.132	92.7	7.3
Weighted	-0.001 (0.082)	0.076	96.9	3.2	0.000 (0.089)	0.090	95.2	4.8	0.000 (0.101)	0.133	88.9	11.1
MR-Egger	-0.004 (0.317)	0.335	92.7	7.3	-0.009 (0.477)	0.496	92.7	7.3	-0.006 (0.646)	0.661	93.0	7.0
Scenario 3. Directional pleiotropy, InSIDE satisfied												
IVW	0.064 (0.089)	0.064	94.8	5.2	0.194 (0.126)	0.076	76.0	24.0	0.388 (0.154)	0.089	16.1	83.9
Robust	0.010 (0.069)	0.064	94.3	5.7	0.069 (0.113)	0.083	93.9	6.1	0.335 (0.227)	0.105	63.6	36.4
Penalized	0.007 (0.062)	0.063	94.2	5.8	0.033 (0.067)	0.078	89.2	10.8	0.148 (0.082)	0.137	57.3	42.7
R and P	0.005 (0.072)	0.065	94.8	5.2	0.025 (0.092)	0.079	93.2	6.7	0.115 (0.138)	0.147	78.6	20.9
Lasso	0.006 (0.063)	0.065	94.2	5.8	0.031 (0.071)	0.080	90.3	9.7	0.164 (0.096)	0.146	60.5	39.5
Median												
Simple	0.021 (0.089)	0.077	97.3	2.7	0.074 (0.100)	0.086	92.9	7.2	0.224 (0.134)	0.124	64.3	35.7
Weighted	0.017 (0.082)	0.074	96.9	3.1	0.065 (0.090)	0.085	91.7	8.3	0.210 (0.110)	0.149	56.9	43.1
MR-Egger	-0.003 (0.318)	0.334	92.9	7.2	-0.001 (0.450)	0.465	93.2	6.8	-0.004 (0.544)	0.562	92.4	7.6
Scenario 4. Directional pleiotropy, InSIDE violated												
IVW	0.077 (0.070)	0.058	83.4	16.7	0.186 (0.075)	0.056	25.6	74.4	0.290 (0.071)	0.050	0.3	99.7
Robust	0.031 (0.085)	0.069	93.9	6.0	0.142 (0.127)	0.082	73.1	26.0	0.289 (0.079)	0.053	3.3	96.6
Penalized	0.021 (0.061)	0.070	89.2	10.8	0.083 (0.063)	0.091	64.1	35.9	0.231 (0.061)	0.092	12.2	87.8
R and P	0.018 (0.071)	0.070	92.7	7.3	0.075 (0.084)	0.095	76.2	23.7	0.230 (0.074)	0.101	19.0	80.8
Lasso	0.024 (0.062)	0.073	88.2	11.8	0.116 (0.066)	0.099	51.1	48.9	0.286 (0.066)	0.070	2.2	97.8
Median												
Simple	0.020 (0.089)	0.077	97.3	2.7	0.071 (0.092)	0.083	89.9	10.1	0.192 (0.088)	0.091	40.0	60.0
Weighted	0.055 (0.082)	0.077	91.0	9.0	0.198 (0.081)	0.097	34.8	65.2	0.343 (0.069)	0.074	0.5	99.5
MR-Egger	0.305 (0.214)	0.219	66.8	33.2	0.539 (0.197)	0.183	21.3	78.7	0.644 (0.182)	0.165	5.1	94.9

Abbreviations: IV, instrumental variable; Est. estimate; SE, standard error; SD, standard deviation; Cov., coverage; Pow., power; InSIDE, instrument strength independent of direct effect; IVW, inverse variance weighted method; R and P, robust and penalized.

**Table 4:** Mean estimate (mean standard error), standard deviation, coverage of the 95% confidence interval (%), and power at the 5% significance level (%) of the estimates from the IVW model with: 1) the *J* genetic variants (IVW); 2) robust regression; 3) penalized weights; and 4) robust regression and penalized weights (R and P) for Scenarios 2-4 with a positive causal effect (*θ* = 0.3) by the number of invalid IVs. Results from the Lasso penalization method with the heterogeneity stopping rule, simple median, weighted median and MR-Egger methods are also provided.

	1 invalid IV				3 invalid IVs				6 invalid IVs			
	Est. (SE)	SD	Cov.	Pow.	Est. (SE)	SD	Cov.	Pow.	Est. (SE)	SD	Cov.	Pow.
**Scenario 2**. Balanced pleiotropy, InSIDE satisfied												
IVW	0.286 (0.097)	0.100	94.0	80.9	0.288 (0.139)	0.140	93.5	54.5	0.285 (0.184)	0.184	93.3	34.3
Robust	0.287 (0.084)	0.079	93.9	91.0	0.288 (0.116)	0.107	93.6	71.1	0.286 (0.193)	0.178	93.8	34.5
Penalized	0.289 (0.074)	0.079	93.2	96.5	0.291 (0.080)	0.098	88.6	91.8	0.295 (0.090)	0.147	78.4	80.5
R and P	0.289 (0.083)	0.080	93.5	92.5	0.290 (0.100)	0.097	92.2	81.6	0.295 (0.145)	0.147	88.1	59.4
Lasso	0.287 (0.076)	0.080	93.2	95.4	0.288 (0.085)	0.102	89.4	88.0	0.288 (0.108)	0.167	80.5	69.9
Median												
Simple	0.302 (0.109)	0.097	97.5	83.1	0.302 (0.118)	0.113	96.1	75.3	0.303 (0.136)	0.155	92.5	61.4
Weighted	0.276 (0.100)	0.091	96.1	81.9	0.277 (0.106)	0.108	94.0	75.6	0.277 (0.119)	0.152	88.2	63.2
MR-Egger	0.143 (0.349)	0.363	90.2	9.0	0.138 (0.495)	0.518	91.2	8.4	0.126 (0.657)	0.681	92.1	7.5
**Scenario 3**. Directional pleiotropy, InSIDE satisfied												
IVW	0.353 (0.098)	0.075	96.1	97.8	0.482 (0.133)	0.087	81.5	99.3	0.673 (0.160)	0.101	28.3	100
Robust	0.306 (0.084)	0.077	95.1	94.8	0.383 (0.134)	0.099	93.9	86.2	0.631 (0.205)	0.112	60.8	90.8
Penalized	0.303 (0.074)	0.078	93.8	98.0	0.346 (0.081)	0.100	86.4	97.8	0.511 (0.098)	0.164	47.6	99.1
R and P	0.300 (0.083)	0.080	94.1	93.5	0.335 (0.102)	0.102	91.3	88.8	0.485 (0.142)	0.179	66.2	86.9
Lasso	0.301 (0.076)	0.079	94.0	97.4	0.340 (0.086)	0.104	88.2	96.5	0.513 (0.113)	0.168	53.8	98.5
Median												
Simple	0.329 (0.110)	0.095	97.5	89.5	0.393 (0.125)	0.108	93.5	93.2	0.572 (0.158)	0.150	61.8	97.2
Weighted	0.300 (0.100)	0.090	97.2	88.1	0.356 (0.111)	0.104	94.5	93.0	0.516 (0.131)	0.161	63.6	97.4
MR-Egger	0.142 (0.345)	0.353	90.9	8.7	0.138 (0.468)	0.485	91.3	8.1	0.137 (0.555)	0.576	91.9	8.0
**Scenario 4**. Directional pleiotropy, InSIDE violated												
IVW	0.367 (0.080)	0.071	88.8	99.8	0.478 (0.084)	0.067	42.2	100	0.582 (0.078)	0.062	2.3	100
Robust	0.329 (0.100)	0.082	94.3	90.2	0.447 (0.128)	0.085	73.7	90.2	0.581 (0.087)	0.066	8.2	99.7
Penalized	0.323 (0.072)	0.086	88.6	98.2	0.403 (0.072)	0.102	61.2	98.9	0.546 (0.068)	0.092	11.2	99.9
R and P	0.318 (0.085)	0.087	92.4	94.1	0.397 (0.095)	0.107	72.7	93.6	0.547 (0.077)	0.098	16.0	98.4
Lasso	0.323 (0.073)	0.089	87.9	97.6	0.430 (0.076)	0.105	52.7	99.3	0.579 (0.073)	0.079	4.4	100
Median												
Simple	0.328 (0.108)	0.095	97.0	89.9	0.387 (0.111)	0.101	90.0	95.1	0.509 (0.101)	0.101	43.4	99.5
Weighted	0.344 (0.099)	0.095	94.0	94.4	0.496 (0.097)	0.108	47.2	99.7	0.625 (0.085)	0.087	3.4	100
MR-Egger	0.488 (0.254)	0.259	86.1	51.8	0.767 (0.233)	0.220	45.4	90.0	0.887 (0.214)	0.197	20.2	98.1

Abbreviations: IV, instrumental variable; Est. estimate; SE, standard error; SD, standard deviation; Cov., coverage; Pow., power; InSIDE, instrument strength independent of direct effect; IVW, inverse variance weighted method; R and P, robust and penalized.

In Scenario 3 (directional pleiotropy, InSIDE satisfied), the IVW method produced biased causal estimates with inflated Type I error rates, and the degree of bias increased with the number of invalid IVs. With one invalid instrument, estimates from the robust methods were only slightly biased and Type I error rates were fairly well controlled. As the number of instruments increased, bias in the estimates for the robust methods also increased, although the magnitude of bias was smaller than the IVW method, and Type I error inflation was less severe. Robust regression with penalized weights performed reasonably well when there was 1 or 3 invalid instruments. Although the median methods give unbiased estimates asymptotically (that is, as the number of participants increases), when pleiotropic effects are directional there is some bias with a finite sample.

In Scenario 4 (directional pleiotropy, InSIDE violated), all of the robust methods produced biased estimates. When there were only one invalid instrument, the magnitude of bias from the robust methods was less severe than the IVW method, and this was particularly true for robust regression with penalized weights. As the number of invalid IVs increased, the performance of the robust methods worsened, and there was little advantage in applying the robust methods compared to the median estimator in Scenario 4 when 6 of the 15 genetic variants were invalid IVs. In this scenario, bias is greater for the weighted median than for the simple median method as the invalid genetic variants are on average more strongly associated with the risk factor than the valid ones. This is because invalid variants are associated with the risk factor directly and via their effect on the confounder. In practical applications, invalid genetic variants will not necessarily be more strongly associated with the risk factor than valid ones, and so the simple median will not necessarily perform better than the weighted median method.

While results were fairly similar for most of the methods, results from the MR-Egger method were often quite different. This is because the other methods are fairly similar in their assumptions (that most genetic variants are valid IVs) and their mode of operation (variants with causal estimates that differ from the consensus are penalized or downweighted). This highlights the importance in an applied analysis of performing a range of methods that make different assumptions, rather than multiple methods that make similar assumptions [43].

Results from applying robust regression and penalized weights to the MR-Egger method are provided in Table D in the S2 Appendix. Although we had hoped that the combination of the MR-Egger method and approaches to reduce the influence of outlying variants would be synergistic in improving robustness, findings were disappointing, and all of the models were affected by the violation of the NOME assumption. A reason for this is the flexibility of the method: in allowing the intercept to differ from zero and allowing outliers that deviate from the regression model, the method permits the IV assumptions to be violated in quite a broad way. In a substantial number of cases, the method identified the wrong variants as invalid, finding an incorrect configuration of valid and invalid variants that appeared to fit the data better.

Finally, results from the one-sample setting are provided in Tables E and F in the S2 Appendix. Bias in the direction of the observational association was observed for all methods. As with the two–sample setting, the median estimators and robust regression with penalized weights produced the least biased estimates, and the IVW with penalized weights was the most precise.

### Increased number of genetic variants

Since many of the methods described in this paper are based on asymptotic theory, it was anticipated that there would be an improvement in the performance of the methods when the data were generated with a larger number of genetic variants. We therefore repeated the simulation study for Scenarios 2–4 for 1 000 simulated datasets with the number of genetic variants increased from 15 to 100, and the number of invalid IVs increased from 1, 3 and 6 to 5, 15 and 30. The bounds of the uniform distribution used to generate the genetic associations with the risk factor ($\beta_{X_{j}}$) were multiplied by $\sqrt{\frac{15}{100}}$ to ensure the average R^2^ values were comparable with the original simulation study. The IVW model with: 1) the full set of genetic variants; 2) robust regression; 3) penalized weights; and 4) robust regression and penalized weights were all applied to the dataset. The Lasso penalization method with the heterogeneity stopping rule was also considered.

#### Results

The mean R^2^ statistic, F-statistic, and *I*^2^ statistic are contained in Table G in the S2 Appendix for Scenarios 2–4 for the null and positive causal effect by the number of invalid IVs. The mean R^2^ values for the 100 genetic variants were slightly higher than the values reported in the original simulation study (Table A in the S2 Appendix). For all of the scenarios considered, there was a significant reduction in the mean F-statistic and *I*^2^ statistic, and we therefore expected the estimates to be affected by weak instrument bias.

Results from the simulation study for the IVW model with: 1) the *J* genetic variants (IVW); 2) robust regression; 3) penalized weights; and 4) robust regression and penalized weights, and the Lasso penalization method with the heterogeneity stopping rule are provided in Table 5.

**Table 5:** Results from the simulation study when 100 genetic variants were simulated for 1 000 datasets. Mean estimate (mean standard error), standard deviation, coverage of the 95% confidence interval (%), and power at the 5% significance level (%) of the estimates from the IVW model with: 1) the *J* genetic variants (IVW); 2) robust regression; 3) penalized weights; and 4) robust regression and penalized weights (R and P) for Scenarios 2-4 with a null causal effect (*θ* = 0) and positive causal effect (*θ* = 0.3) by the number of invalid instrumental variables. Results from the Lasso penalization method with the heterogeneity stopping rule are also presented.

	5 invalid IV				15 invalid IVs				30 invalid IVs			
	Est. (SE)	SD	Cov.	Pow.	Est. (SE)	SD	Cov.	Pow.	Est. (SE)	SD	Cov.	Pow.
**Null causal effect (*θ* = 0)**												
**Scenario 2**. Balanced pleiotropy, InSIDE satisfied												
IVW	-0.003 (0.072)	0.071	95.0	5.0	-0.003 (0.103)	0.105	94.9	5.1	0.000 (0.138)	0.144	94.0	6.0
Robust	-0.001 (0.054)	0.051	95.8	4.2	-0.001 (0.065)	0.066	93.7	6.3	0.005 (0.115)	0.114	95.7	4.3
Penalized	-0.001 (0.051)	0.051	94.8	5.2	-0.001 (0.054)	0.063	91.3	8.7	0.001 (0.060)	0.081	86.3	13.7
R and P	-0.001 (0.055)	0.052	95.8	4.2	-0.001 (0.064)	0.062	95.6	4.4	0.000 (0.087)	0.078	96.5	3.5
Lasso	-0.001 (0.051)	0.052	94.2	5.8	-0.002 (0.055)	0.063	91.5	8.5	0.002 (0.062)	0.081	87.6	12.4
**Scenario 3**. Directional pleiotropy, InSIDE satisfied												
IVW	0.096 (0.071)	0.058	77.3	22.7	0.287 (0.099)	0.070	9.0	91.0	0.572 (0.126)	0.088	0.0	100
Robust	0.014 (0.055)	0.053	94.8	5.2	0.070 (0.072)	0.064	87.0	13.0	0.355 (0.169)	0.103	41.3	58.7
Penalized	0.012 (0.051)	0.053	93.9	6.1	0.043 (0.054)	0.061	83.9	16.1	0.156 (0.062)	0.094	34.3	65.7
R and P	0.009 (0.055)	0.054	95.2	4.7	0.031 (0.064)	0.061	91.7	8.3	0.108 (0.087)	0.093	74.6	25.4
Lasso	0.013 (0.051)	0.054	93.2	6.8	0.044 (0.055)	0.062	83.4	16.6	0.165 (0.063)	0.095	32.6	67.4
**Scenario 4**. Directional pleiotropy, InSIDE violated												
IVW	0.170 (0.052)	0.046	7.2	92.8	0.349 (0.049)	0.043	0.0	100	0.476 (0.043)	0.036	0.0	100
Robust	0.076 (0.079)	0.065	87.2	12.8	0.310 (0.089)	0.057	7.7	92.1	0.475 (0.048)	0.038	0.0	100
Penalized	0.053 (0.049)	0.064	72.9	27.1	0.187 (0.046)	0.082	13.5	86.5	0.401 (0.040)	0.062	0.0	100
R and P	0.047 (0.058)	0.064	84.5	15.5	0.184 (0.065)	0.087	26.7	73.3	0.409 (0.044)	0.062	0.0	100
Lasso	0.072 (0.048)	0.068	62.9	37.1	0.276 (0.043)	0.072	0.7	99.3	0.474 (0.035)	0.051	0.0	100
**Positive causal effect (*θ* = 0.3)**												
**Scenario 2**. Balanced pleiotropy, InSIDE satisfied												
IVW	0.227 (0.079)	0.076	17.8	82.2	0.229 (0.108)	0.113	45.5	54.5	0.228 (0.141)	0.136	64.2	35.8
Robust	0.227 (0.065)	0.062	6.1	93.9	0.233 (0.080)	0.082	18.6	81.4	0.230 (0.126)	0.119	54.3	45.7
Penalized	0.230 (0.061)	0.062	3.9	96.1	0.241 (0.064)	0.079	8.2	91.8	0.241 (0.071)	0.100	15.7	84.3
R and P	0.229 (0.064)	0.063	4.8	95.2	0.237 (0.072)	0.077	11.1	88.9	0.236 (0.088)	0.097	25.5	74.5
Lasso	0.227 (0.061)	0.064	4.8	95.2	0.232 (0.065)	0.079	9.2	90.8	0.230 (0.072)	0.095	17.8	82.2
**Scenario 3**. Directional pleiotropy, InSIDE satisfied												
IVW	0.323 (0.079)	0.068	1.1	98.9	0.514 (0.107)	0.081	0	100	0.804 (0.133)	0.098	0.0	100
Robust	0.251 (0.066)	0.066	2.9	97.1	0.342 (0.093)	0.081	1.3	98.7	0.654 (0.162)	0.107	0.2	99.8
Penalized	0.251 (0.061)	0.066	2.1	97.9	0.308 (0.065)	0.080	0.8	99.2	0.490 (0.076)	0.121	0.0	100
R and P	0.246 (0.065)	0.067	4.1	95.9	0.291 (0.074)	0.080	3.2	96.8	0.442 (0.102)	0.123	1.6	98.3
Lasso	0.247 (0.061)	0.066	2.5	97.5	0.297 (0.065)	0.081	1.3	98.7	0.463 (0.074)	0.115	0.0	100
**Scenario 4**. Directional pleiotropy, InSIDE violated												
IVW	0.411 (0.061)	0.060	0.0	100	0.609 (0.058)	0.054	0.0	100	0.747 (0.051)	0.045	0.0	100
Robust	0.327 (0.095)	0.076	4.5	95.5	0.575 (0.093)	0.065	0.6	99.4	0.746 (0.057)	0.047	0.0	100
Penalized	0.307 (0.058)	0.080	0.9	99.1	0.479 (0.053)	0.093	0.1	99.9	0.689 (0.046)	0.066	0.0	100
R and P	0.298 (0.072)	0.080	1.7	98.3	0.478 (0.073)	0.098	0.2	99.6	0.697 (0.051)	0.066	0.0	100
Lasso	0.314 (0.057)	0.08	0.3	99.7	0.544 (0.050)	0.081	0.0	100	0.742 (0.041)	0.061	0.0	100

Abbreviations: IV, instrumental variable; Est. estimate; SE, standard error; SD, standard deviation; Cov., coverage; Pow., power; InSIDE, instrument strength independent of direct effect; IVW, inverse variance weighted; R and P, robust regression and penalized weights.

The reduction in the strength of the IVs led to weak instrument bias, and there was severe attenuation towards the null for the positive causal effect (Table 5). For the null causal effect, there was little difference in the performance of the robust methods with the increased number of genetic variants. In fact, the methods performed worst under Scenario 4 when 100 variants were included in the data generating model rather than 15 (Table 5). Due to the attenuation of the positive causal effect when the number of variants was increased to 100, it was difficult to compare the results to the original simulations. Nevertheless, there was no evidence to suggest that the performances of the robust methods improved when the number of genetic variants was increased.

## Discussion

In this paper, we have introduced three robust approaches for Mendelian randomization with summary level data that downweight the influence of heterogeneous causal estimates. The applied examples considered in this paper illustrate the importance of using a variety of methods in a Mendelian randomization analysis. The results from the robust methods support a null causal effect of BMI on schizophrenia risk. While the IVW and MR-Egger methods produced positive estimates that were strongly influenced by pleiotropic variants in the *APOE* gene region, the proposed methods were able to give null estimates that were unaffected by these outlying variants.

We also performed a simulation study to compare the robust approaches to the IVW, simple median, weighted median, and MR-Egger methods. The simulation study highlighted the sensitivity of the IVW method to violations in the IV assumptions, and the requirement for robust methods to be considered in the sensitivity analysis of a Mendelian randomization study. The simulations also demonstrated the impact of violating the NOME assumption on the estimates from the MR-Egger methods. Since it was not feasible to adjust for the violation of the NOME assumption through the SIMEX method [42] in the simulation study for computational reasons, it was difficult to compare the performance of the robust methods to MR-Egger.

Robust regression with penalized weights consistently produced the least biased estimates in the simulation study. Although the power and bias of this approach was significantly better than the standard IVW method when the IV assumptions were violated, it suffered from poor coverage and increased Type I error rates, particularly when there was a high proportion of invalid instruments. When there was only one invalid instrument, robust regression with penalized weights produced more precise estimates than the median estimator. However, as the number of invalid instruments increased there was little advantage of using robust regression with penalized weights compared to the median estimator.

### Interpretation of heterogeneity among the causal ratio estimates

Throughout this paper, we have assumed that heterogeneity of the causal ratio estimates is indicative of violations in the IV assumptions, particularly the presence of pleiotropic effects. However, heterogeneity among the causal ratio estimates may arise for a number of reasons [44]. For example, there may be multiple mechanisms of intervention on a complex risk factor, each of which has an associated causal effect. For a two-sample Mendelian randomization analysis, there may be heterogeneity among the causal ratio estimates due to substantial differences in the study populations used to estimate the genetic associations with the risk factor and outcome. The robust approaches considered in this paper penalize genetic variants with heterogeneous causal ratio estimates regardless of how this heterogeneity has materialised. As such, these methods should only be employed if it is suspected that the IV assumptions have been violated, and other possible reasons for heterogeneity among the causal ratio estimates explored.

### Issues with penalizing genetic variants

The simulation study has highlighted some of the disadvantages of excluding or downweighting genetic variants from Mendelian randomization analyses. Excluding genetic variants with heterogeneous causal estimates will generally reduce the standard error of the estimate. However, too much penalization can potentially result in artificial overconfidence in the precision of the causal estimate, leading to poor coverage of the true causal effect and increased Type I error rates, as seen for Lasso penalization. If the excluded genetic variants are truly invalid IVs then removing them from the analysis will reduce bias and improve the precision of the causal estimate. However, outlying or heterogeneous causal ratio estimates may be valid IVs, and so removing them from the analysis would be inappropriate. On balance, it may be more appropriate to consider approaches that reduce the contribution that heterogeneous ratio estimates have on the causal estimate, such as the median estimator or robust regression, rather than excluding them from the analysis. If a large number of variants are identified as outliers, then researchers should consider reporting that the Mendelian randomization analysis is inconclusive, rather than reporting a causal estimate.

### Implication for Mendelian randomization studies

The purpose of this paper was not to promote one robust method for Mendelian randomization over another, but to emphasize the need for multiple sensitivity analyses that make different sets of assumptions. Although we acknowledge that none of the proposed methods performed significantly better than the median estimator, the extensions proposed in this paper should provide additional confidence in the findings from a conventional Mendelian randomization analysis, particularly when the causal estimates are consistent. Genetic variants that are downweighted or excluded from the analysis by the robust methods should be examined for pleiotropy to determine whether they should be removed from the dataset. The methods proposed here are likely to be useful for Mendelian randomization analyses performed for large numbers of risk factors in an automated manner, such as for -omics risk factors measured on a high-throughput platform. These methods can help a researcher rapidly triage whether a positive causal estimate from the standard IVW method is evidenced just by a small number of variants (as in the LDL-cholesterol and Alzheimer’s disease example), or by the majority of variants.

The methods introduced in this paper, particularly robust regression with penalized weights, may be more suited to certain scenarios than the median estimator. In the applied example for LDL-C and AD risk, there were two variants that appeared to be clear outliers. The median estimator and robust regression with penalized weights both suggested that there was a null causal effect of LDL-C on AD risk, but the estimates from the median estimator were less precise. This observation of robust regression producing more precise estimates was also observed in the simulation study when there was one invalid IV. Robust regression with penalized weights may be a useful addition to sensitivity analyses in Mendelian randomization when there are a small proportion of variants with heterogeneous causal estimates.

### Limitations

We found that the Lasso penalization method may be more appropriate in an applied setting, where the estimates can be reported over a range of values of the tuning parameter. The practicality of applying Lasso penalization to the simulation study was more restrictive, and required an automated approach to selecting the tuning parameter.

Whilst we appreciate the limitation of only considering methods with uncorrelated genetic variants, we argue that robust methods should be used when the IV assumptions are in doubt, and therefore using one genetic variant from each gene region is a sensible (although conservative) approach for robust methods in an applied Mendelian randomization analysis. This is because including multiple variants from a single region may mean that region receives a disproportionate weight in the analysis, and so the validity of the analysis would be overly dependent on the validity of these variants. This could be problematic as correlated variants are likely to all be valid or all be invalid, particularly if they are all in the same gene region. If an analyst does want to include correlated variants in an analysis, this can be done by first calculating the appropriate weighting matrix based on the inverse-variance weights and the correlations between variants, and multiplying the genetic associations by the Cholesky decomposition of this matrix, as described previously [45]. Software code to do this is provided in S1 Appendix. However, we caution that no allowance is made that correlated variants are likely to all be valid or all invalid simultaneously, as the methods treat all association estimates as separate datapoints.

The violation of the NOME assumption limited the utility of the simulation study as the estimates from MR-Egger could not be compared to the robust methods. Given that MR-Egger is frequently used as part of a sensitivity analysis in Mendelian randomization studies, this could be viewed as a weakness of the simulation study.

The main simulation study was also limited by the number of genetic variants considered in the data generating model. Since GWASs are now being performed on large study populations, and estimates of genetic associations are publicly available from large consortia, only considering 15 variants in the simulation study may have been conservative. We tried to rectify this limitation by re-performing the simulation study with 100 genetic variants (but keeping the overall *R*^2^ statistic similar) and found that there was significant attenuation towards the null due to weak instrument bias. We had thought that the performances of some of the robust methods would have improved by increasing the number of genetic variants as the methods are based on asymptotic theory. However, we did not find any significant improvements in the methods, and in some cases, the performance of the models worsened with the increased number of genetic variants.

## Conclusion

This paper has highlighted the difficulty in robust causal inference when genetic variants in a Mendelian randomization analysis violate the IV assumptions. The extensions proposed in this paper are by no means perfect; even when a small proportion of the variants were invalid IVs, all methods had inflated Type I error rates in at least one scenario. Nevertheless, the Type I error rate for the proposed extensions was substantially better than the IVW method and MR-Egger when the InSIDE assumption was violated.

This paper has demonstrated the benefits of using multiple robust methods as part of a sensitivity analysis. We suggest that the IVW method using robust regression with penalized weights may be a worthwhile additional sensitivity analysis to be performed in a Mendelian randomization analysis in addition to previously proposed methods.

## Supporting information

S1 Appendix Software code. R code for performing the approaches outlined in the paper, and extracting genetic association estimates.
(PDF)

S2 Appendix Supplementary tables from the simulation study. Additional results from the simulation study.
(PDF)

## References

[1] Davey Smith G, Ebrahim S. ‘Mendelian randomization’: can genetic epidemiology contribute to understanding environmental determinants of disease? International Journal of Epidemiology. 2003;32(1):1–22. 12689998 10.1093/ije/dyg070

[2] Lawlor DA, Harbord RM, Sterne JA, Timpson N, Davey Smith G. Mendelian randomization: using genes as instruments for making causal inferences in epidemiology. Statistics in Medicine. 2008;27(8):1133–1163. 10.1002/sim.3034 17886233

[3] Greenland S. An introduction to instrumental variables for epidemiologists. International Journal of Epidemiology. 2000;29(4):722–729. 10.1093/ije/29.4.722 10922351

[4] Burgess S, Scott RA, Timpson NJ, Davey Smith G, Thompson SG. Using published data in Mendelian randomization: a blueprint for efficient identification of causal risk factors. European Journal of Epidemiology. 2015;30(7):543–552. 10.1007/s10654-015-0011-z 25773750 PMC4516908

[5] Burgess S, Butterworth A, Thompson SG. Mendelian randomization analysis with multiple genetic variants using summarized data. Genetic Epidemiology. 2013;37(7):658–665. 10.1002/gepi.21758 24114802 PMC4377079

[6] Angrist JD, Imbens GW. Two-stage least squares estimation of average causal effects in models with variable treatment intensity. Journal of the American Statistical Association. 1995;90(430):431–442. 10.1080/01621459.1995.10476535

[7] Burgess S, Thompson SG. Use of allele scores as instrumental variables for Mendelian randomization. International Journal of Epidemiology. 2013;42(4):1134–1144. 10.1093/ije/dyt093 24062299 PMC3780999

[8] Bowden J, Davey Smith G, Haycock PC, Burgess S. Consistent Estimation in Mendelian Randomization with Some Invalid Instruments Using a Weighted Median Estimator. Genetic Epidemiology. 2016;40(4):304–314. 10.1002/gepi.21965 27061298 PMC4849733

[9] Bowden J, Davey Smith G, Burgess S. Mendelian randomization with invalid instruments: effect estimation and bias detection through Egger regression. International Journal of Epidemiology. 2015;44(2):512–525. 10.1093/ije/dyv080 26050253 PMC4469799

[10] Verbanck M, Chen CY, Neale B, Do R. Detection of widespread horizontal pleiotropy in causal relationships inferred from Mendelian randomization between complex traits and diseases. Nature Genetics. 2018;50(5):693–698. 10.1038/s41588-018-0099-7 29686387 PMC6083837

[11] Dai JY, Peters U, Wang X, Kocarnik J, Chang-Claude J, Slattery ML, et al Diagnostics for Pleiotropy in Mendelian Randomization Studies: Global and Individual Tests for Direct Effects. American Journal of Epidemiology. 2018;187(12):2672–2680. 10.1093/aje/kwy177 30188971 PMC6269243

[12] Zhu Z, Zheng Z, Zhang F, Wu Y, Trzaskowski M, Maier R, et al Causal associations between risk factors and common diseases inferred from GWAS summary data. Nature Communications. 2018;9(1):224 10.1038/s41467-017-02317-2 29335400 PMC5768719

[13] Del Greco M F, Minelli C, Sheehan NA, Thompson JR. Detecting pleiotropy in Mendelian randomisation studies with summary data and a continuous outcome. Statistics in Medicine. 2015;34(21):2926–2940. 10.1002/sim.6522 25950993

[14] Bowden J, Del Greco M F, Minelli C, Zhao Q, Lawlor DA, Sheehan NA, et al Improving the accuracy of two-sample summary-data Mendelian randomization: moving beyond the NOME assumption. International Journal of Epidemiology. 2018. 10.1093/ije/dyy258 PMC6659376 30561657

[15] Huber P. Robust Statistics. Wiley; 2009.

[16] Yavorska OO, Burgess S. MendelianRandomization: an R package for performing Mendelian randomization analyses using summarized data. International Journal of Epidemiology. 2017 10.1093/ije/dyx034 28398548 PMC5510723

[17] Burgess S, CCGC (CHD CRP Genetics Collaboration). Identifying the odds ratio estimated by a two-stage instrumental variable analysis with a logistic regression model. Statistics in Medicine. 2013;32(27):4726–4747. 10.1002/sim.5871 23733419 PMC3935453

[18] Vansteelandt S, Bowden J, Babanezhad M, Goetghebeur E. On instrumental variables estimation of causal odds ratios. Statistical Science. 2011;26(3):403–422. 10.1214/11-STS360

[19] Kolesár M, Chetty R, Friedman J, Glaeser E, Imbens G. Identification and inference with many invalid instruments. Journal of Business & Economic Statistics. 2015;33(4):474–484. 10.1080/07350015.2014.978175

[20] Koller M, Stahel W. Sharpening wald-type inference in robust regression for small samples. Computational Statistics & Data Analysis. 2011;55(8):2504–2515. 10.1016/j.csda.2011.02.014

[21] Rousseeuw P, Croux C, Todorov V, Ruckstuhl A, Salibian-Barrera M, Verbeke T, et al. robustbase: Basic Robust Statistics; 2015 URL http://cran.r-project.org/package=robustbase, R package version 0.92-5.

[22] Bowden J, Hemani G, Davey Smith G. Invited Commentary: Detecting Individual and Global Horizontal Pleiotropy in Mendelian Randomization—A Job for the Humble Heterogeneity Statistic? American Journal of Epidemiology. 2018;187(12):2681–2685. 10.1093/aje/kwy185 30188969 PMC6269239

[23] Rucker G, Schwarzer G, Carpenter JR, Binder H, Schumacher M. Treatment effect estimates adjusted for small-study effects via a limit meta-analysis. Biostatistics. 2011;12(1):122–142. 10.1093/biostatistics/kxq046 20656692

[24] Kang H, Zhang A, Cai T, Small D. Instrumental Variables Estimation With Some Invalid Instruments and its Application to Mendelian Randomization. Journal of the American Statistical Association. 2016;111(513):132–144. 10.1080/01621459.2014.994705

[25] Windmeijer F, Farbmacher H, Davies N, Davey Smith G. On the Use of the Lasso for Instrumental Variables Estimation with Some Invalid Instruments. Journal of the American Statistical Association. 2018;0(0):1–12. 10.1080/01621459.2018.1498346 PMC6817329 31708716

[26] Cheng X, Liao Z. Select the valid and relevant moments: An information-based LASSO for GMM with many moments. Journal of Econometrics. 2015;186(2):443–464. 10.1016/j.jeconom.2015.02.019

[27] Tibshirani R. Regression Shrinkage and Selection Via the Lasso. Journal of the Royal Statistical Society, Series B (Methodological). 1996;58:267–288. 10.1111/j.2517-6161.1996.tb02080.x

[28] Goeman J, Meijer R, Chaturvedi N, Lueder M. penalized: L1 (Lasso and Fused Lasso) and L2 (Ridge) Penalized Estimation in GLMs and in the Cox Model; 2017 URL https://cran.r-project.org/web/packages/penalized/penalized.pdf.

[29] Staley JR, Blackshaw J, Kamat MA, Ellis S, Surendran P, Sun BB, et al PhenoScanner: a database of human genotype-phenotype associations. Bioinformatics. 2016;32(20):3207–3209. 10.1093/bioinformatics/btw373 27318201 PMC5048068

[30] Coodin S. Body mass index in persons with schizophrenia. Canadian Journal of Psychiatry. 2001;46(6):549–555. 10.1177/070674370104600610 11526812

[31] Allison DB, Fontaine KR, Heo M, Mentore JL, Cappelleri JC, Chandler LP, et al The distribution of body mass index among individuals with and without schizophrenia. Journal of Clinical Psychiatry. 1999;60(4):215–220. 10.4088/jcp.v60n0402 10221280

[32] Locke AE, Kahali B, Berndt SI, Justice AE, Pers TH, Day FR, et al Genetic studies of body mass index yield new insights for obesity biology. Nature. 2015;518(7538):197–206. 10.1038/nature14177 25673413 PMC4382211

[33] Ripke S, Neale BM, Corvin A, Walters JT, Farh KH, Holmans PA, et al Biological insights from 108 schizophrenia-associated genetic loci. Nature. 2014;511(7510):421–427. 10.1038/nature13595 25056061 PMC4112379

[34] Hartwig FP, Bowden J, Loret de Mola C, Tovo-Rodrigues L, Davey Smith G, Horta BL. Body mass index and psychiatric disorders: a Mendelian randomization study. Scientific Reports. 2016;6:32730 10.1038/srep32730 27601421 PMC5013405

[35] Notkola IL, Sulkava R, Pekkanen J, Erkinjuntti T, Ehnholm C, Kivinen P, et al Serum total cholesterol, apolipoprotein E epsilon 4 allele, and Alzheimer’s disease. Neuroepidemiology. 1998;17(1):14–20. 10.1159/000026149 9549720

[36] Solomon A, Kareholt I, Ngandu T, Winblad B, Nissinen A, Tuomilehto J, et al Serum cholesterol changes after midlife and late-life cognition: twenty-one-year follow-up study. Neurology. 2007;68(10):751–756. 10.1212/01.wnl.0000256368.57375.b7 17339582

[37] Shepardson NE, Shankar GM, Selkoe DJ. Cholesterol level and statin use in Alzheimer disease: I. Review of epidemiological and preclinical studies. JAMA Neurology. 2011;68(10):1239–1244. 10.1001/archneurol.2011.203 PMC3211071 21987540

[38] Do R, Willer CJ, Schmidt EM, Sengupta S, Gao C, Peloso GM, et al Common variants associated with plasma triglycerides and risk for coronary artery disease. Nature Genetics. 2013;45(11):1345–1352. 10.1038/ng.2795 24097064 PMC3904346

[39] Willer CJ, Schmidt EM, Sengupta S, Peloso GM, Gustafsson S, Kanoni S, et al Discovery and refinement of loci associated with lipid levels. Nature Genetics. 2013;45(11):1274–1283. 10.1038/ng.2797 24097068 PMC3838666

[40] Benn M, Nordestgaard BG, Frikke-Schmidt R, Tybjærg-Hansen A. Low LDL cholesterol, PCSK9 and HMGCR genetic variation, and risk of Alzheimer’s disease and Parkinson’s disease: Mendelian randomisation study. British Medical Journal. 2017;357 10.1136/bmj.j1648 28438747 PMC5421439

[41] Lambert JC, Ibrahim-Verbaas CA, Harold D, Naj AC, Sims R, Bellenguez C, et al Meta-analysis of 74,046 individuals identifies 11 new susceptibility loci for Alzheimer’s disease. Nature Genetics. 2013;45(12):1452–1458. 10.1038/ng.2802 24162737 PMC3896259

[42] Bowden J, Del Greco M F, Minelli C, Davey Smith G, Sheehan NA, Thompson JR. Assessing the suitability of summary data for two-sample Mendelian randomization analyses using MR-Egger regression: the role of the I^2^ statistic. International Journal of Epidemiology. 2016;45(6):1961–1974. 10.1093/ije/dyw220 27616674 PMC5446088

[43] Slob EA, Burgess S. A comparison of robust Mendelian randomization methods using summary data. bioRxiv. 2019; p. 577940. 10.1002/gepi.22295 PMC7317850 32249995

[44] Hemani G, Bowden J, Davey Smith G. Evaluating the potential role of pleiotropy in Mendelian randomization studies. Human Molecular Genetics. 2018;27(R2):R195–R208. 10.1093/hmg/ddy163 29771313 PMC6061876

[45] Burgess S, Thompson SG. Interpreting findings from Mendelian randomization using the MR-Egger method. European Journal of Epidemiology. 2017;32(5):377–389. 10.1007/s10654-017-0255-x 28527048 PMC5506233

