# Supplementary material for: Robust methods in Mendelian randomization via penalization of heterogeneous causal estimates
Source: PLoS One. 2019 Sep 23;14(9):e0222362. doi: 10.1371/journal.pone.0222362 (PMC6756542; doi:10.1371/journal.pone.0222362)
Supplement: S1 Appendix — R code for performing the approaches outlined in the paper, and extracting genetic association estimates. (PDF) [file pone.0222362.s001.pdf]

S1 Software code

We provide R code to implement the methods discussed in this paper. The associations of the genetic variants with the exposure are denoted `betaXG` with standard errors `sebetaXG`. The associations of the genetic variants with the outcome are denoted `betaYG` with standard errors `sebetaYG`. We assume that the genetic variants are uncorrelated.

Inverse-variance weighted estimate

The inverse-variance weighted (IVW) estimate can be calculated by weighted linear regression:

```
betaIVW              = summary(lm(betaYG~betaXG-1, weights=sebetaYG^-2))$coef[1]
sebetaIVW.fixed      = summary(lm(betaYG~betaXG-1, weights=sebetaYG^-2))$coef[1,2]/
                       summary(lm(betaYG~betaXG-1, weights=sebetaYG^-2))$sigma
sebetaIVW.random     = summary(lm(betaYG~betaXG-1, weights=sebetaYG^-2))$coef[1,2]/
                       min(summary(lm(betaYG~betaXG-1, weights=sebetaYG^-2))$sigma,1)
```

In the fixed-effect model, we divide the reported standard error by the estimated residual standard error to force the residual standard error to be 1. In the multiplicative random-effects model, we divide by the estimated residual standard error when the variability in the genetic associations is less than expected by chance (underdispersion). When there is evidence of heterogeneity between the causal estimates (overdispersion) the standard error is unaltered. The multiplicative random-effects model will result in a larger standard error compared to the fixed-effect model if there is heterogeneity between the causal estimates. The point estimate is unaffected by the choice of a fixed- or multiplicative random-effects model.

Alternatively, the inverse-variance weighted estimate can be calculated by meta-analysis, or via a simple formula:

```
# meta-analysis
library(meta)
betaIVW              = metagen(betaYG/betaXG, abs(sebetaYG/betaXG))$TE.fixed
sebetaIVW.fixed      = metagen(betaYG/betaXG, abs(sebetaYG/betaXG))$seTE.fixed
# simple formula
betaIVW              = sum(betaYG*betaXG*sebetaYG^-2)/sum(betaXG^2*sebetaYG^-2)
sebetaIVW.fixed      = 1/sqrt(sum(betaXG^2*sebetaYG^-2))
```

MR-Egger regression

The MR-Egger method is equivalent to the IVW method calculated using weighted regression, except that that intercept term is estimated rather than being set to zero. A test as to whether the intercept term is equal to zero is a test of directional pleiotropy and the validity of the InSIDE assumption. The genetic associations with the risk factor `betaXG` and outcome `betaYG` must be orientated with respect to the risk increasing or decreasing allele of the risk factor. A random-effects model should be used for inference as a fixed-effect model is not justifiable when the genetic variants are not all valid instruments.

```
# coding of genetic variants
betaYG = betaYG*sign(betaXG); betaXG = abs(betaXG)
# causal estimate
betaEGGER           = summary(lm(betaYG~betaXG, weights=sebetaYG^-2))$coef[2,1]
sebetaEGGER.random  = summary(lm(betaYG~betaXG, weights=sebetaYG^-2))$coef[2,2]/
                      min(summary(lm(betaYG~betaXG, weights=sebetaYG^-2))$sigma, 1)
betaEGGER.lower     = betaEGGER-qt(0.975,df=length(betaXG)-2)*sebetaEGGER.random
betaEGGER.upper     = betaEGGER+qt(0.975,df=length(betaXG)-2)*sebetaEGGER.random
p.causal.random     = 2*(1-pt(abs(betaEGGER/sebetaEGGER.random),df=length(betaXG)-2))
# test for directional pleiotropy
interEGGER          = summary(lm(betaYG~betaXG, weights=sebetaYG^-2))$coef[1,1]
seinterEGGER.random = summary(lm(betaYG~betaXG, weights=sebetaYG^-2))$coef[1,2]/
                      min(summary(lm(betaYG~betaXG, weights=sebetaYG^-2))$sigma, 1)
p.dpleio.random     = 2*(1-pt(abs(interEGGER/seinterEGGER.random),df=length(betaXG)-2))
```

In this code, we use a t-distribution with $J-2$ degrees of freedom for inference. If there is underdispersion, then the t-distribution may be overly conservative, as the t-distribution assumes that the residual standard error is estimated (in case of under-dispersion, the residual standard error is set to 1). Hence, if the residual standard error is less than one, either a confidence interval using a residual standard error of 1 and a z-distribution, or else a confidence interval using the estimated residual standard error and a t-distribution may be preferred (the wider of these two intervals should be preferred – both of these will be narrower than the above confidence interval).

```
sigmaEGGER         = summary(lm(betaYG~betaXG, weights=sebetaYG^-2))$sigma
betaEGGER.lower    = ifelse(sigmaEGGER<1, min(betaEGGER-qnorm(0.975)*sebetaEGGER.random,
                     betaEGGER-qt(0.975,df=length(betaXG)-2)*sebetaEGGER.random*sigmaEGGER),
                     betaEGGER-qt(0.975,df=length(betaXG)-2)*sebetaEGGER.random)
betaEGGER.upper    = ifelse(sigmaEGGER<1, max(betaEGGER+qnorm(0.975)*sebetaEGGER.random,
                     betaEGGER+qt(0.975,df=length(betaXG)-2)*sebetaEGGER.random*sigmaEGGER),
                     betaEGGER+qt(0.975,df=length(betaXG)-2)*sebetaEGGER.random)
```

Median-based method

The median-based method calculates the median (or weighted median) of the causal estimates from each candidate instrument. This code calculates the simple median and weighted median, employing bootstrapping to obtain a standard error that can be used to provide a confidence interval.

```
weighted.median <- function(betaIV.in, weights.in) {
  betaIV.order  = betaIV.in[order(betaIV.in)]
  weights.order = weights.in[order(betaIV.in)]
  weights.sum   = cumsum(weights.order)-0.5*weights.order
  weights.sum   = weights.sum/sum(weights.order)
        below   = max(which(weights.sum<0.5))
  weighted.est  = betaIV.order[below] + (betaIV.order[below+1]-betaIV.order[below])*
                  (0.5-weights.sum[below])/(weights.sum[below+1]-weights.sum[below])
  return(weighted.est) }
 #
weighted.median.boot = function(betaXG.in, betaYG.in, sebetaXG.in, sebetaYG.in, weights.in){
 # the standard error is estimated based on 1000 bootstrap samples
med = NULL
for(i in 1:1000){
 betaXG.boot = rnorm(length(betaXG.in), mean=betaXG.in, sd=sebetaXG.in)
 betaYG.boot = rnorm(length(betaYG.in), mean=betaYG.in, sd=sebetaYG.in)
 betaIV.boot = betaYG.boot/betaXG.boot
 med[i]      = weighted.median(betaIV.boot, weights.in)
 }
return(sd(med)) }
 #
```

```
betaIV              = betaYG/betaXG
weights             = rep(1, length(betaXG)) # unweighted median
betaSIMPLEMED       = weighted.median(betaIV, weights)
sebetaSIMPLEMED     = weighted.median.boot(betaXG, betaYG, sebetaXG, sebetaYG, weights)
lowerSIMPLEMED      = betaSIMPLEMED-qnorm(0.975)*sebetaSIMPLEMED
upperSIMPLEMED      = betaSIMPLEMED+qnorm(0.975)*sebetaSIMPLEMED
 #
betaIV              = betaYG/betaXG
weights             = (sebetaYG/betaXG)^-2   # weighted median using inverse-variance weights
betaWEIGHTEDMED     = weighted.median(betaIV, weights)
sebetaWEIGHTEDMED   = weighted.median.boot(betaXG, betaYG, sebetaXG, sebetaYG, weights)
lowerWEIGHTEDMED    = betaWEIGHTEDMED-qnorm(0.975)*sebetaWEIGHTEDMED
upperWEIGHTEDMED    = betaWEIGHTEDMED+qnorm(0.975)*sebetaWEIGHTEDMED
```

Robust regression

The IVW and MR-Egger methods can be performed using robust regression (in particular, MM-estimation using Tukey's bisquare objective function) rather than standard linear regression:

```
library(robustbase)
betaIVW.robust            = summary(lmrob(betaYG~betaXG-1, weights=sebetaYG^-2, k.max=500))$coef[1]
sebetaIVW.robust.fixed    = summary(lmrob(betaYG~betaXG-1, weights=sebetaYG^-2, k.max=500))$coef[1,2]/
                            summary(lmrob(betaYG~betaXG-1, weights=sebetaYG^-2, k.max=500))$sigma
sebetaIVW.robust.random   = summary(lmrob(betaYG~betaXG-1, weights=sebetaYG^-2, k.max=500))$coef[1,2]/
                            min(summary(lmrob(betaYG~betaXG-1, weights=sebetaYG^-2, k.max=500))$sigma,1)
betaEGGER.robust          = summary(lmrob(betaYG~betaXG,   weights=sebetaYG^-2, k.max=500))$coef[2]
sebetaEGGER.robust.random = summary(lmrob(betaYG~betaXG,   weights=sebetaYG^-2, k.max=500))$coef[2,2]/
                            min(summary(lmrob(betaYG~betaXG,   weights=sebetaYG^-2, k.max=500))$sigma,1)
```

The `k.max` option sets the maximum number of steps evaluated to find initial parameter values in the S-step of the algorithm. The `lmrob` function sets the tuning parameter to 1.548 to provide a high breakdown point in the S-estimation step, and as 4.685 to provide efficiency in the M-estimation step.

Penalized weights

The IVW and MR-Egger methods can be performed using penalized weights:

```
betaIVW                  = sum(betaYG*betaXG*sebetaYG^-2)/sum(betaXG^2*sebetaYG^-2)
pweights                 = pchisq(betaXG^2/sebetaYG^2*(betaYG/betaXG-betaIVW)^2, df=1, lower.tail=FALSE)
pweightsE                = pchisq(sebetaYG^-2*(betaYG - interEGGER - betaEGGER*betaXG)^2, df=1, lower.tail=FALSE)
rweights                 = sebetaYG^-2*pmin(1, pweights*100)
rweightsE                = sebetaYG^-2*pmin(1, pweightsE*100)
betaIVW.penal            = summary(lm(betaYG~betaXG-1, weights=rweights))$coef[1]
sebetaIVW.penal.fixed    = summary(lm(betaYG~betaXG-1, weights=rweights))$coef[1,2]/
                           summary(lm(betaYG~betaXG-1, weights=rweights))$sigma
sebetaIVW.penal.random   = summary(lm(betaYG~betaXG-1, weights=rweights))$coef[1,2]/
                           min(summary(lm(betaYG~betaXG-1, weights=rweights))$sigma,1)
betaEGGER.penal          = summary(lm(betaYG~betaXG,   weights=rweightsE))$coef[2]
sebetaEGGER.penal.random = summary(lm(betaYG~betaXG,   weights=rweightsE))$coef[2,2]/
                           min(summary(lm(betaYG~betaXG,   weights=rweightsE))$sigma,1)
```

Penalized weights can also be used in conjunction with robust regression.

Lasso penalization

Several packages are available for running Lasso penalization methods. We chose the *penalized* package as this gave an option for some of the coefficients in the model to be penalized (the pleiotropy intercept parameters), and others not to be penalized (the causal effect parameter):

```
library(penalized)
# dividing the association estimates by sebetaYG is equivalent to weighting by
    sebetaYG^-2
betaYGw = betaYG/sebetaYG
betaXGw = betaXG/sebetaYG
pleio   = diag(rep(1, length(betaXG)))

# values of lambda for heterogeneity stopping rule
l1grid = c(seq(from=0.1, to=5, by=0.1), seq(from=5.2, to=10, by=0.2))
l1grid_rse = NULL; l1grid_length = NULL; l1grid_beta = NULL; l1grid_se = NULL

for (i in 1:length(l1grid)) {
 l1grid_which     = which(attributes(penalized(betaYGw, pleio, betaXGw,
                    lambda1=l1grid[i], trace=FALSE))$penalized==0)
 l1grid_rse[i]    = summary(lm(betaYG[l1grid_which]~betaXG[l1grid_which]-1,
                    weights=sebetaYG[l1grid_which]^-2))$sigma
 l1grid_length[i] = length(l1grid_which)
 l1grid_beta[i]   = lm(betaYG[l1grid_which]~betaXG[l1grid_which]-1,
                    weights=sebetaYG[l1grid_which]^-2)$coef[1]
 l1grid_se[i]     = summary(lm(betaYG[l1grid_which]~betaXG[l1grid_which]-1,
                    weights=sebetaYG[l1grid_which]^-2))$coef[1,2]/
                    min(summary(lm(betaYG[l1grid_which]~betaXG[l1grid_which]-1,
                    weights=sebetaYG[l1grid_which]^-2))$sigma, 1)
 }

# heterogeneity criterion for choosing lambda
l1which_hetero = c(which(l1grid_rse[1:(length(l1grid)-1)]>1
                 &diff(l1grid_rse)>qchisq(0.95, df=1)/
                 l1grid_length[2:length(l1grid)]), length(l1grid))[1]
l1hetero_beta  = l1grid_beta[l1which_hetero]
l1hetero_se    = l1grid_se[l1which_hetero]

# cross-validation criterion for choosing lambda
l1xval_lambda  = optL1(betaYGw, pleio, betaXGw)$lambda
l1xval_which   = which(attributes(penalized(betaYGw, pleio, betaXGw,
                 lambda1=l1xval_lambda))$penalized==0)
l1xval_beta    = summary(lm(alpy[l1xval_which]~alpx[l1xval_which]-1,
                 weights=alpysd[l1xval_which]^-2))$coef[1]
l1xval_se      = summary(lm(alpy[l1xval_which]~alpx[l1xval_which]-1,
                 weights=alpysd[l1xval_which]^-2))$coef[1,2]/
                 min(summary(lm(alpy[l1xval_which]~alpx[l1xval_which]-1,
                 weights=alpysd[l1xval_which]^-2))$sigma, 1)
```

We found that our choice of values of λ ($0.1, 0.2, \ldots, 4.9, 5.0, 5.2, 5.4, \ldots, 9.8, 10.0$) worked well in both the simulations and the applied example. However, for different sets of association estimates, a different choice of values may be preferred. Additionally, particularly with large numbers of variants, a more dense choice of values may be preferred to ensure that at most one variant is added to the analysis at each incremental step.

Regression diagnostics

Studentized residuals and Cook's distance can be calculated for the IVW method as:

```
rstudent(lm(betaYG~betaXG-1, weights=sebetaYG^-2))
cooks.distance(lm(betaYG~betaXG-1, weights=sebetaYG^-2))
```

Correlated variants

The IVW method can be performed for correlated variants using the standard linear regression command after weighting the data by the Cholesky decomposition of a weighting matrix `Omega` that accounts for the inverse-variance weights and the correlation between the genetic variants, where `rho` is the (signed) correlation matrix whose (i, j)th element is the correlation between genetic variant i and genetic variant j:

```
Omega   = sebetaYG%o%sebetaYG*rho
c_betaXG = solve(t(chol(Omega)))%*%betaXG
c_betaYG = solve(t(chol(Omega)))%*%betaYG
beta_IVWcorrel  = lm(c_betaYGc_betaXG1)$coef[1]
se_IVWcorrel.fixed  = sqrt(1/(t(betaXG)%*%solve(Omega)%*%betaXG))
se_IVWcorrel.random = sqrt(1/(t(betaXG)%*%solve(Omega)%*%betaXG))*
                      max(summary(lm(c_betaYGc_betaXG1))$sigma,1)
```

The robust and penalized methods can be implemented for correlated variants similarly by replacing the `lm` command with the appropriate function.

Genetic variants and genetic associations

We provide R code to obtain data for the applied examples from PhenoScanner, a database of genetic association estimates. This code is included in the `MendelianRandomization` package for R that is available through The Comprehensive R Archive Network (CRAN) with kind permission of James Staley.

Body mass index and schizophrenia

In total, 97 genetic variants (listed below) were used in the analysis of body mass index and schizophrenia. At the time of writing, PhenoScanner was not able to process quadrallelic genetic variants, and so it only retrieved data on 95 of the variants. It is hoped that this will be fixed in the near future.

```
library(MendelianRandomization)

bmi_snps = scan(what="character")
rs1558902 rs6567160 rs13021737 rs10938397 rs543874 rs2207139 rs11030104 rs3101336 rs7138803
 rs10182181 rs3888190 rs1516725 rs12446632 rs2287019 rs16951275 rs3817334 rs2112347 rs12566985
 rs3810291 rs7141420 rs13078960 rs10968576 rs17024393 rs657452 rs12429545 rs12286929 rs13107325
 rs11165643 rs7903146 rs10132280 rs17405819 rs6091540 rs1016287 rs4256980 rs17094222 rs12401738
 rs7599312 rs2365389 rs205262 rs2820292 rs12885454 rs9641123 rs12016871 rs16851483 rs1167827
 rs758747 rs1928295 rs9925964 rs11126666 rs2650492 rs6804842 rs12940622 rs7164727 rs11847697
 rs4740619 rs492400 rs13191362 rs3736485 rs17001654 rs11191560 rs2080454 rs7715256 rs2176040
 rs1528435 rs2075650 rs1000940 rs2033529 rs11583200 rs7239883 rs2836754 rs9400239 rs10733682
 rs11688816 rs11057405 rs9914578 rs977747 rs2121279 rs29941 rs11727676 rs3849570 rs9374842
 rs6477694 rs4787491 rs1441264 rs7899106 rs2176598 rs2245368 rs17203016 rs17724992 rs7243357
 rs16907751 rs1808579 rs13201877 rs2033732 rs9540493 rs1460676 rs6465468

bmi_obj = pheno_input(snps=bmi_snps,
 exposure = "Body mass index", pmidE = "25673413", ancestryE = "European",
 outcome = "Schizophrenia", pmidO = "25056061", ancestryO = "Mixed")

mr_ivw(bmi_obj)                  # IVW method
mr_egger(bmi_obj)                # MR-Egger method
mr_median(bmi_obj)               # Weighted median method
mr_ivw(bmi_obj, robust = TRUE)    # Robust regression method
mr_ivw(bmi_obj, penalized = TRUE) # Penalized weights method
```

LDL-cholesterol and Alzheimer's disease

In total, 75 genetic variants (listed below) were used in the analysis of LDL-cholesterol and Alzheimer's disease. Data for all these variants are available in PhenoScanner.

```
ldl_snps = scan(what="character")
rs10903129 rs4587594 rs6603981 rs646776 rs1010167 rs267733 rs2642438 rs903319 rs2587534
 rs1367117 rs515135 rs6544713 rs4148218 rs2710642 rs17508045 rs2030746 rs16831243 rs1250229
 rs11563251 rs9875338 rs7640978 rs17345563 rs7703051 rs4530754 rs6882076 rs2294261 rs1800562
 rs2247056 rs868943 rs2297374 rs1564348 rs12670798 rs4722551 rs2073547 rs217386 rs4240624
 rs10102164 rs2326077 rs2737252 rs2980885 rs2954022 rs7832643 rs3780181 rs1883025 rs8176720
 rs579459 rs2255141 rs10832962 rs174532 rs1535 rs10790162 rs11220462 rs653178 rs6489818 rs1169288
 rs4942486 rs8017377 rs9989419 rs2288002 rs2000999 rs314253 rs7225700 rs6511720 rs688 rs10401969
 rs6859 rs7254892 rs492602 rs364585 rs2328223 rs7264396 rs6016381 rs6065311 rs1800961 rs5763662

ldl_obj = pheno_input(snps=ldl_snps,
 exposure = "Low density lipoprotein", pmidE = "24097068", ancestryE = "European",
 outcome = "Alzheimers disease", pmidO = "24162737", ancestryO = "European")

mr_ivw(ldl_obj)                  # IVW method (and so on)
```

The genetic association estimates can also be obtained from the web-based version of PhenoScanner at `http://www.phenoscanner.medschl.cam.ac.uk/`.
