# Supplementary material for: Robust methods in Mendelian randomization via penalization of heterogeneous causal estimates
Source: PLoS One. 2019 Sep 23;14(9):e0222362. doi: 10.1371/journal.pone.0222362 (PMC6756542; doi:10.1371/journal.pone.0222362)
Supplement: S2 Appendix — Additional results from the simulation study. (PDF) [file pone.0222362.s002.pdf]

S2 Supplementary tables from the simulation study

Mean values for the R^2, F-statistic and I^2 statistic

Table A contains the mean R^2 (%), F-statistic and I^2 (%) from the simulation study for all scenarios considered.

Table A. Mean values of the R^2 (%), F-statistic and I^2 (%) for Scenarios 1-4 with a null ($\theta = 0$) or positive ($\theta = 0.3$) causal effect by the number of invalid instrumental variables (IV).

	No invalid IVs			1 invalid IV			3 invalid IVs			6 invalid IVs		
	R^2	F	I^2	R^2	F	I^2	R^2	F	I^2	R^2	F	I^2
**Null causal effect: $\theta = 0$**												
Scenario 1	3.0	20.8	39.6	-	-	-	-	-	-	-	-	
Scenario 2	-	-	-	3.0	20.8	39.6	3.0	20.8	39.3	3.0	20.8	39.5
Scenario 3	-	-	-	3.0	20.8	39.7	3.0	20.8	39.5	3.0	20.8	39.2
Scenario 4	-	-	-	3.4	23.6	56.5	4.2	29.3	70.7	5.4	37.7	77.5
**Positive causal effect: $\theta = 0.3$**												
Scenario 1	3.0	20.8	39.3	-	-	-	-	-	-	-	-	
Scenario 2	-	-	-	3.0	20.8	39.1	3.0	20.8	39.4	3.0	20.8	39.6
Scenario 3	-	-	-	3.0	20.8	39.9	3.0	20.8	39.7	3.0	20.8	39.6
Scenario 4	-	-	-	3.4	23.6	56.4	4.2	29.3	70.8	5.4	37.7	77.4

Number of robust regression analyses without a standard error

The number of robust regressions that did not report a standard error in the simulations are presented in Table B. The proportion of simulations was less than 1.2% across the different scenarios for the IVW model. Apart from the calculation of the mean standard error, the simulations that did not report a standard error were included in the results, and the power calculations treated the standard error as infinite.

Table B. Number of the 10 000 simulations that failed to report a standard error using robust regression (without and with penalized weights) with the inverse-variance weighted (IVW) and MR-Egger methods, for Scenarios 1-4 with a null ($\theta = 0$) or positive ($\theta = 0.3$) causal effect by the number of invalid instrumental variables.

	IVW								MR-Egger							
	Robust				Robust, penalized				Robust				Robust, penalized			
No. invalid:	0	1	3	6	0	1	3	6	0	1	3	6	0	1	3	6
**Null causal effect: $\theta = 0$**																
Scenario 1	0	-	-	-	0	-	-	-	16	-	-	-	16	-	-	-
Scenario 2	-	1	2	5	-	3	9	120	-	24	72	78	-	45	98	258
Scenario 3	-	2	1	4	-	3	10	51	-	32	69	32	-	30	70	139
Scenario 4	-	3	84	11	-	5	6	22	-	144	100	5	-	124	76	9
**Positive causal effect: $\theta = 0.3$**																
Scenario 1	4	-	-	-	3	-	-	-	13	-	-	-	13	-	-	-
Scenario 2	-	0	0	1	-	1	3	54	-	20	55	47	-	24	71	211
Scenario 3	-	0	0	3	-	3	2	22	-	24	72	19	-	37	62	73
Scenario 4	-	2	30	4	-	0	9	9	-	151	91	10	-	122	81	15

Abbreviations: IVW, inverse-variance weighted; No., number.

MR-Egger intercept test

Table C contains information on the power (at the 5% significance level) of the intercept test in the MR-Egger method for detecting directional pleiotropy and/or violation of the InSIDE assumption for all scenarios.

Table C. Power (%) of the intercept test in the MR-Egger method for detecting directional pleiotropy and/or violation of the InSIDE assumption for Scenarios 1-4 with a null ($\theta = 0$) or positive ($\theta = 0.3$) causal effect by the number of invalid instrumental variables (IV).

	Null causal effect				Positive causal effect			
No. invalid:	0	1	3	6	0	1	3	6
Scenario 1	3.7	-	-	-	8.7	-	-	
Scenario 2	-	7.2	7.5	7.0	-	9.4	8.5	7.8
Scenario 3	-	7.2	8.7	13.1	-	11.2	13.8	19.1
Scenario 4	-	22.8	49.9	55.9	-	8.6	26.2	32.0

Results from applying the robust methods to the MR-Egger method

Table D contains the results from the simulation study when the MR-Egger model was applied to the simulated data with: 1) robust regression (R); 2) penalized weights (P); and 3) robust regression and penalized weights (R and P).

Table D. Mean (standard error) estimates and power from the MR-Egger method with: robust regression (R); penalized weights (P); and robust regression and penalized weights (R and P) for Scenarios 1-4 with a null ($\theta = 0$) or positive ($\theta = 0.3$) causal effect by the number of invalid instrumental variables.

	No invalid IVs		1 invalid IV		3 invalid IVs		6 invalid IVs	
	Mean (mean SE)	Power, %	Mean (mean SE)	Power, %	Mean (mean SE)	Power, %	Mean (mean SE)	Power, %
**Null causal effect: $\theta = 0$**								
Scenario 1. No pleiotropy, InSIDE satisfied								
R	0.000 (0.231)	8.2	-	-	-	-	-	-
P	-0.001 (0.216)	4.3	-	-	-	-	-	-
R and P	0.000 (0.230)	8.3	-	-	-	-	-	-
Scenario 2. Balanced pleiotropy, InSIDE satisfied								
R	-	-	-0.006 (0.245)	9.7	-0.002 (0.375)	9.6	-0.007 (0.671)	10.8
P	-	-	-0.006 (0.208)	9.9	-0.003 (0.231)	16.9	-0.009 (0.274)	31.5
R and P	-	-	-0.007 (0.254)	9.2	-0.001 (0.333)	10.7	-0.008 (0.505)	20.3
Scenario 3. Directional pleiotropy, InSIDE satisfied								
R	-	-	-0.003 (0.246)	9.9	0.001 (0.376)	9.5	-0.004 (0.564)	13.8
P	-	-	-0.004 (0.208)	10.1	0.001 (0.249)	18.7	-0.009 (0.343)	37.9
R and P	-	-	-0.004 (0.256)	9.4	0.001 (0.309)	12.0	-0.005 (0.419)	32.4
Scenario 4. Directional pleiotropy, InSIDE violated								
R	-	-	0.171 (0.291)	18.2	0.493 (0.234)	65.1	0.649 (0.158)	95.6
P	-	-	0.241 (0.196)	33.0	0.527 (0.178)	81.2	0.651 (0.159)	97.7
R and P	-	-	0.173 (0.272)	18.5	0.490 (0.215)	68.2	0.652 (0.148)	96.8
**Positive causal effect: $\theta = 0.3$**								
Scenario 1. No pleiotropy, InSIDE satisfied								
R	0.144 (0.273)	13.1	-	-	-	-	-	-
P	0.143 (0.258)	7.9	-	-	-	-	-	-
R and P	0.144 (0.271)	13.3	-	-	-	-	-	-
Scenario 2. Balanced pleiotropy, InSIDE satisfied								
R	-	-	0.140 (0.295)	13.1	0.139 (0.430)	11.6	0.124 (0.665)	11.9
P	-	-	0.139 (0.255)	13.5	0.140 (0.282)	19.4	0.130 (0.331)	30.2
R and P	-	-	0.140 (0.297)	12.9	0.140 (0.363)	14.6	0.133 (0.508)	22.4
Scenario 3. Directional pleiotropy, InSIDE satisfied								
R	-	-	0.141 (0.295)	13.2	0.135 (0.433)	11.8	0.136 (0.563)	14.7
P	-	-	0.140 (0.252)	13.2	0.137 (0.302)	19.9	0.137 (0.392)	30.7
R and P	-	-	0.140 (0.292)	13.3	0.135 (0.352)	15.9	0.138 (0.433)	31.0
Scenario 4. Directional pleiotropy, InSIDE violated								
R	-	-	0.338 (0.340)	25.5	0.719 (0.274)	75.6	0.893 (0.190)	97.7
P	-	-	0.418 (0.233)	48.9	0.754 (0.210)	91.2	0.895 (0.188)	99.4
R and P	-	-	0.340 (0.319)	27.1	0.716 (0.249)	78.6	0.897 (0.179)	98.2

Abbreviations: IV, instrumental variable; SE, standard error; InSIDE, instrument strength independent of direct effect; R, robust regression; P, penalized weights.

Results from the one-sample setting

Results from the simulation study when the data were generated from one sample are contained in Table E (null causal effect $\theta = 0$) and Table F (positive causal effect $\theta = 0.3$). Estimates from the IVW model with: 1) the J genetic variants (IVW); 2) robust regression (R); 3) penalized weights (P); and 4) robust regression and penalized weights (R and P), and the Lasso penalization (LP) method with the heterogeneity stopping rule for Scenarios 1-4 are displayed in the Tables E and F.

Table E. Mean (standard error) and power (%) of the estimates from the IVW model with: 1) the J genetic variants (IVW); 2) robust regression (R); 3) penalized weights (P); and 4) robust regression and penalized weights (R and P) for Scenarios 1-4 with a null causal effect ($\theta = 0$) by the number of invalid instrumental variables for one-sample Mendelian randomization. Results from the Lasso penalization (LP) method with the heterogeneity stopping rule are also provided.

	No invalid IVs		1 invalid IV		3 invalid IVs		6 invalid IVs	
	Mean (mean SE)	Pow., %	Mean (mean SE)	Pow., %	Mean (mean SE)	Pow., %	Mean (mean SE)	Pow., %
**Null causal effect: $\theta = 0$**								
Scenario 1. No pleiotropy, InSIDE satisfied								
IVW	0.021 (0.061)	5.6	-	-	-	-	-	-
R	0.021 (0.065)	6.8	-	-	-	-	-	-
P	0.019 (0.060)	6.0	-	-	-	-	-	-
R and P	0.020 (0.063)	7.2	-	-	-	-	-	-
LP	0.021 (0.060)	6.2	-	-	-	-	-	-
Scenario 2. Balanced pleiotropy, InSIDE satisfied								
IVW	-	-	0.020 (0.088)	6.4	0.020 (0.132)	7.1	0.024 (0.180)	7.3
R	-	-	0.021 (0.068)	7.8	0.020 (0.096)	6.8	0.023 (0.195)	5.9
P	-	-	0.018 (0.062)	7.1	0.015 (0.066)	9.7	0.008 (0.075)	19.7
R and P	-	-	0.019 (0.070)	6.8	0.017 (0.092)	6.3	0.010 (0.156)	7.7
LP	-	-	0.020 (0.063)	7.1	0.020 (0.070)	9.2	0.019 (0.088)	16.9
Scenario 3. Directional pleiotropy, InSIDE satisfied								
IVW	-	-	0.086 (0.088)	9.3	0.216 (0.123)	36.2	0.409 (0.150)	92.5
R	-	-	0.032 (0.067)	8.8	0.088 (0.109)	11.2	0.357 (0.222)	44.9
P	-	-	0.025 (0.062)	7.0	0.046 (0.067)	13.6	0.132 (0.081)	40.4
R and P	-	-	0.025 (0.070)	7.5	0.040 (0.088)	10.8	0.103 (0.125)	21.5
LP	-	-	0.027 (0.063)	7.2	0.049 (0.071)	12.6	0.173 (0.096)	42.8
Scenario 4. Directional pleiotropy, InSIDE violated								
IVW	-	-	0.096 (0.068)	27.5	0.202 (0.072)	86.6	0.303 (0.067)	100.0
R	-	-	0.053 (0.081)	10.1	0.163 (0.119)	38.4	0.302 (0.072)	98.4
P	-	-	0.040 (0.061)	14.2	0.095 (0.062)	41.4	0.237 (0.061)	89.6
R and P	-	-	0.038 (0.069)	11.4	0.089 (0.079)	30.7	0.236 (0.071)	83.9
LP	-	-	0.048 (0.062)	17.2	0.138 (0.066)	58.8	0.300 (0.064)	99.1

Abbreviations: IV, instrumental variable; SE, standard error; Pow., power; InSIDE, instrument strength independent of direct effect; IVW, inverse variance weighted; R, robust regression; P, penalized weights; LP, lasso penalization.

Table F. Mean (standard error) and power (%) of the estimates from the IVW model with: 1) the J genetic variants (IVW); 2) robust regression (R); 3) penalized weights (P); and 4) robust regression and penalized weights (R and P) for Scenarios 1-4 with a positive causal effect ($\theta = 0.3$) by the number of invalid instrumental variables for one-sample Mendelian randomization. Results from the Lasso penalization (LP) method with the heterogeneity stopping rule are also provided.

	No invalid IVs		1 invalid IV		3 invalid IVs		6 invalid IVs	
	Mean (mean SE)	Pow., %	Mean (mean SE)	Pow., %	Mean (mean SE)	Pow., %	Mean (mean SE)	Pow., %
**Positive causal effect: $\theta = 0.3$**								
Scenario 1. No pleiotropy, InSIDE satisfied								
IVW	0.321 (0.068)	99.7	-	-	-	-	-	-
R	0.321 (0.073)	97.7	-	-	-	-	-	-
P	0.321 (0.068)	99.7	-	-	-	-	-	-
R and P	0.321 (0.072)	97.9	-	-	-	-	-	-
LP	0.321 (0.068)	99.7	-	-	-	-	-	-
Scenario 2. Balanced pleiotropy, InSIDE satisfied								
IVW	-	-	0.322 (0.090)	91.2	0.322 (0.133)	66.2	0.323 (0.180)	43.9
R	-	-	0.322 (0.073)	97.2	0.321 (0.098)	86.1	0.323 (0.193)	43.6
P	-	-	0.320 (0.070)	99.1	0.316 (0.076)	96.4	0.308 (0.087)	85.4
R and P	-	-	0.321 (0.078)	96.3	0.317 (0.096)	88.4	0.310 (0.138)	67.3
LP	-	-	0.322 (0.071)	99.2	0.320 (0.079)	95.6	0.320 (0.099)	81.4
Scenario 3. Directional pleiotropy, InSIDE satisfied								
IVW	-	-	0.386 (0.088)	99.7	0.517 (0.124)	100.0	0.710 (0.150)	100.0
R	-	-	0.332 (0.073)	97.9	0.390 (0.111)	93.3	0.655 (0.226)	86.8
P	-	-	0.330 (0.070)	99.7	0.362 (0.076)	99.5	0.465 (0.093)	99.5
R and P	-	-	0.328 (0.078)	96.0	0.351 (0.093)	92.6	0.434 (0.125)	89.1
LP	-	-	0.331 (0.071)	99.6	0.363 (0.079)	99.4	0.508 (0.108)	99.2
Scenario 4. Directional pleiotropy, InSIDE violated								
IVW	-	-	0.396 (0.070)	100.0	0.502 (0.073)	100.0	0.601 (0.067)	100.0
R	-	-	0.352 (0.088)	95.5	0.463 (0.125)	89.7	0.601 (0.073)	99.7
P	-	-	0.349 (0.068)	99.7	0.424 (0.068)	99.7	0.561 (0.064)	99.9
R and P	-	-	0.343 (0.078)	97.0	0.414 (0.090)	95.7	0.562 (0.071)	98.8
LP	-	-	0.357 (0.068)	99.7	0.463 (0.070)	99.8	0.600 (0.066)	100.0

Abbreviations: IV, instrumental variable; SE, standard error; Pow., power; InSIDE, instrument strength independent of direct effect; IVW, inverse variance weighted; R, robust regression; P, penalized weights; LP, lasso penalization.

Mean values for the R^2, F-statistic and I^2 statistic for 100 genetic variants

Table G contains the mean R^2 (%), F-statistic and I^2 (%) when the simulation study was re-performed for 100 genetic variants for Scenarios 2-4.

Table G. Mean values of the R^2 (%), F-statistic and I^2 (%) for Scenarios 2-4 with a null ($\theta = 0$) or positive ($\theta = 0.3$) causal effect by the number of invalid instrumental variables (IV) when the simulation study was re-perfomed for 100 genetic variants.

	5 invalid IV			15 invalid IVs			30 invalid IVs		
	R^2	F	I^2	R^2	F	I^2	R^2	F	I^2
**Null causal effect: $\theta = 0$**									
Scenario 2	4.0	4.2	3.0	4.0	4.2	3.3	4.0	4.2	2.9
Scenario 3	4.0	4.2	3.1	4.0	4.2	3.0	4.0	4.2	3.0
Scenario 4	5.2	5.4	32.9	7.3	7.8	58.3	10.3	11.4	69.5
**Positive causal effect: $\theta = 0.3$**									
Scenario 2	4.0	4.2	3.2	4.0	4.2	3.1	4.1	4.2	3.1
Scenario 3	4.0	4.2	3.3	4.1	4.2	3.1	4.0	4.2	2.9
Scenario 4	5.2	5.4	33.3	7.3	7.8	58.2	10.3	11.4	69.4
